# Multi‐Resonant Thermally Activated Delayed Fluorescent (MR‐TADF) Compounds as Photocatalysts**

**DOI:** 10.1002/chem.202202998

**Published:** 2022-11-14

**Authors:** Callum Prentice, James Morrison, Andrew D. Smith, Eli Zysman‐Colman

**Affiliations:** ^1^ Organic Semiconductor Centre EaStCHEM School of Chemistry University of St Andrews St Andrews Fife KY169ST UK; ^2^ EaStCHEM School of Chemistry University of St Andrews St Andrews Fife KY169ST UK; ^3^ Pharmaceutical Sciences, IMED Biotech Unit AstraZeneca Macclesfield SK102NA UK

**Keywords:** kinetics, multi-resonant thermally activated delayed fluorescence (MR-TADF), photocatalysis, thermally activated delayed fluorescence (TADF)

## Abstract

Donor‐acceptor (D−A) thermally activated delayed fluorescent (TADF) compounds, such as **4CzIPN**, have become a widely used sub‐class of organic photocatalysts for a plethora of photocatalytic reactions. Multi‐resonant TADF (MR‐TADF) compounds, a subclass of TADF emitters that are rigid nanographene derivatives, such as **DiKTa** and **Mes_3_DiKTa**, have to date not been explored as photocatalysts. In this study both **DiKTa** and **Mes_3_DiKTa** were found to give comparable or better product yield than **4CzIPN** in a range of photocatalytic processes that rely upon reductive quenching, oxidative quenching, energy transfer and dual photocatalytic processes. In a model oxidative quench process, **DiKTa** and **Mes_3_DiKTa** gave increased reaction rates in comparison to **4CzIPN**, with **DiKTa** being of particular interest due to the lower material cost (£0.94/mmol) compared to that of **4CzIPN** (£3.26/mmol). These results suggest that **DiKTa** and **Mes_3_DiKTa** would be excellent additions to any chemist's collection of photocatalysts.

## Graphical abstract

**Multi‐resonant thermally** activated delayed fluorescence (MR‐TADF) compounds, **DiKTa** and **Mes_3_DiKTa**, are shown to be excellent photocatalysts in a range of different reactions, benchmarked against the widely used donor‐acceptor TADF photocatalyst, **4CzIPN**. Advantages of using these MR‐TADF photocatalysts include robust and inexpensive photocatalyst synthesis, lower required photocatalyst loadings and faster reaction rates, while achieving comparable or improved product yields.

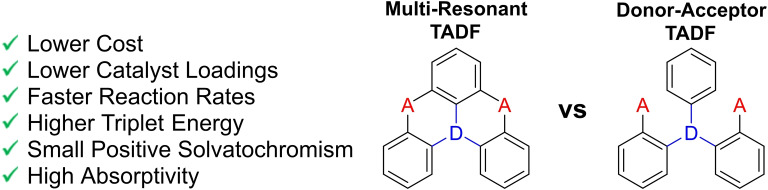

## Introduction

**Figure 1:**
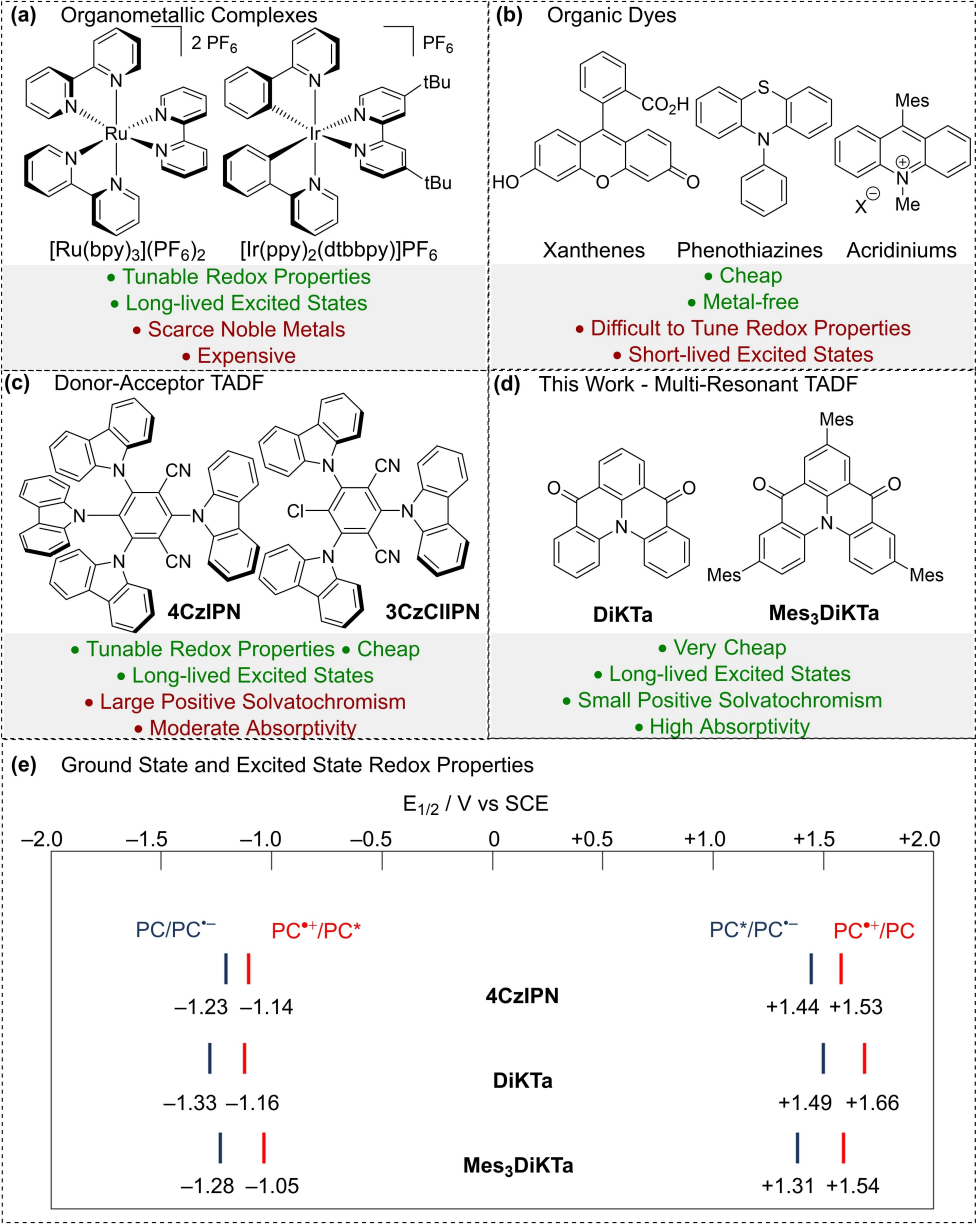
(a) Examples of organometallic PCs. (b) Examples of organic PCs. (c) Examples of D−A TADF PCs. (d) MR‐TADF photocatalysts used in this work. (e) Excited state and ground state redox potentials of **4CzIPN** in MeCN.^[14]^ Ground state redox potentials of **DiKTa** and **Mes_3_DiKTa** in MeCN.^[15]^ Excited state redox properties of **DiKTa** and **Mes_3_DiKTa** calculated from the experimentally determined E_ox_/E_red_ and E_0,0_ values in MeCN, using E_ox_(PC⋅^+^/PC*)=E_ox_−E_0,0_ and E_red_(PC*/PC⋅^−^)=E_red_+E_0,0_.^[15]^

Visible‐light photocatalysis has become a widely used technique in synthetic organic chemistry. Photocatalysis functions by harnessing the electronic excited state of a photocatalyst (PC*), generated via the absorption of a photon, to interact with an organic substrate through either electron or energy transfer. Photocatalytic photoinduced single electron transfer (PET), commonly known as photoredox catalysis, proceeds either via a reductive or an oxidative quenching mechanism, dependent on whether the PC* gains or loses an electron, respectively, during the initial PET.^[1]^ Alternatively, photoinduced energy transfer (PEnT) implicates the transfer of energy from the PC* to the substrate through either a Dexter or Förster energy transfer mechanism, regenerating the ground state photocatalyst (PC).^[2]^ Organometallic complexes based on Ru(II) and Ir(III) are the most widely used PCs (Figure 1a). They possess an attractive suite of properties including suitably long‐lived stable excited states, absorption that extends into the visible region where most organic substrates are transparent, plus (especially for Ir(III) complexes), the capacity to modulate both the ground and excited state redox properties through ligand variation.^[3]^ However, the scarcity, toxicity and cost of the noble metals employed has spurred intense efforts to find alternative PCs. There are now many established examples of Earth‐abundant metal complexes^[4]^ and metal‐free organic photocatalysts,^[5]^ and numerous examples where these perform comparably to the noble metal PCs. While organic photocatalysts, such as xanthene dyes, phenothiazines, and acridinium‐based compounds are commonplace (Figure 1b),[^5^, ^6^, ^7^, ^8^] their ground and excited state redox potentials are difficult to tune. Donor‐acceptor (D−A) thermally activated delayed fluorescent (TADF) PCs, most widely exemplified by the compound **4CzIPN**, have rapidly been adopted in the field as their properties are readily tuneable through substituent variation (Figure 1c).[^9^, ^10^] **4CzIPN**, initially developed as an emitter for organic light‐emitting diodes a decade ago, luminesces via a TADF mechanism.^[11]^ As a result, **4CzIPN** possesses microsecond‐long emission lifetimes that, coupled with similar redox properties to that of the widely used [Ir(dF(CF_3_)ppy)_2_(dtbbpy)]PF_6_, endows it with similar photochemical reactivity. **4CzIPN** was first used as a photocatalyst by Luo and Zhang to achieve a dual catalysed (C)sp^3^−(C)sp^2^ cross‐coupling reaction.^[12]^ Subsequent work by Speckmeier et al.^[13]^ demonstrated the versatility of this class of D−A TADF photocatalyst and the tunability of their redox potentials by varying the nature and number of electron‐donating and electron‐accepting groups. A growing number of structurally related D−A PCs have since been reported.^[16]^ TADF operates when the energy gap between the lowest energy singlet and triplet excited states, Δ*E_ST_*, is sufficiently small such that there is an endothermic upconversion of triplet excitons into singlets by reverse intersystem crossing (RISC). This is possible when the exchange integral between the frontier orbitals involved in the emissive excited state is sufficiently small, which occurs in D−A compounds where the donor and acceptor groups are poorly conjugated such as when they adopt a highly twisted conformation, as is the case for 4CzIPN and its derivatives. An alternative molecular design strategy to reduce the exchange integral is based on the exploitation of opposing resonance effects of p‐ and n‐dopants in nanographenes that is embodied in multi‐resonance TADF (MR‐TADF) emitters.^[17]^ Herein we present the use of two MR‐TADF compounds as photocatalysts for the first time, using DiKTa and Mes_3_DiKTa, previously reported by us as emitters in OLEDs,^[15]^ as typical examples (Figure 1d). Owing to their rigid structure, MR‐TADF compounds typically show much narrower emission profiles and smaller Stokes shifts while also exhibiting larger molar absorptivities for the low‐energy short‐range charge transfer (SRCT) absorption band (see Figure S2). The emissive excited state also shows SRCT character, which is identifiable due to the modest positive solvatochromism, in contrast to the large positive solvatochromism observed for D−A TADF compounds (see Figures S3–S4).^[15]^ Enhanced molar absorptivity and reduced positive solvatochromism are expected to have positive implications for photocatalysis reactivity. The higher molar absorptivity of the band that is being targeted for photoexcitation could translate to faster reaction rates and lower required photocatalyst loadings. The attenuated positive solvatochromism of MR‐TADF compounds implies that less energy is lost due to stabilization of the excited state by solvent, potentially leading to greater reactivity of the PC, particularly in commonly used polar aprotic solvents such as MeCN and DMF. DiKTa and its mesitylated analogue Mes_3_DiKTa were chosen for investigation as photocatalysts because of their similar redox potentials to those of 4CzIPN (Figure 1e).[^13^, ^15^] An additional benefit is that the raw material cost per mmol is significantly lower for DiKTa (£0.94/mmol) than for 4CzIPN (£3.26/mmol, see Supporting Information). These PCs were assessed across a diverse range of transformations including reductive quenching reactions, oxidative quenching reactions, energy transfer reactions, nickel dual catalysis and hydrogen atom transfer (HAT) dual catalysis. The result of this assessment shows that DiKTa and Mes_3_DiKTa are attractive alternatives to the widely used 4CzIPN.

## Results and Discussion

### Reductive quench

Our investigations began with a decarboxylative photo‐Giese reaction. This process has previously been reported by Ji et al.^[8]^ for their comparison of the effectiveness of different acridinium photocatalysts and also by Speckmeier et al.^[13]^ for their comparison of the suitability of alternative D−A photocatalysts. In the latter study Speckmeier et al. found that when **4CzIPN** was used, a superior isolated yield of 80 % is achieved compared to the previously reported best acridinium photocatalyst, which produced an isolated yield of 73 %. Using *N*‐Cbz protected proline **1 a** as the carboxylic acid substrate and diethyl maleate **2** as the electron deficient alkene, both **DiKTa** and **Mes_3_DiKTa** gave comparable NMR yields to that of **4CzIPN** (Table 1, entries 1–3). *N*‐protected prolines have relatively low oxidation potentials [E_ox_([Boc‐Pro‐O][/Cs])=0.95 V vs. SCE in MeCN];^[18]^ therefore, the more challenging *iso*‐butyric acid, **1 b**, and propanoic acid, **1 c**, ([E_ox_[*i*PrCO_2_][/NBu_4_])=1.31 V and E_ox_([EtCO_2_][/NBu_4_])=1.25 V)^[19]^ were also investigated in order to differentiate the photooxidation ability of the PCs. With *iso‐*butyric acid both **DiKTa** and **Mes_3_DiKTa** showed improved NMR yields of 78 % and 79 %, respectively (Table 1, entries 4 and 5), relative to the 64 % achieved using **4CzIPN** (Table 1, entry 6). Changing to the primary radical formed when using propanoic acid resulted in lower yields for all three PCs (Table 1, entries 7–9). This is likely due to the decreased nucleophilicity of primary radicals relative to secondary radicals, leading to alternative and undesired reaction pathways becoming competitive. Notwithstanding the lower yields, both **DiKTa** and **Mes_3_DiKTa** still outperformed **4CzIPN** (Table 1, entries 7–9). **4CzIPN** in the presence of similar carboxylic acids has been shown by König et al.^[14]^ to undergo photosubstitution with the radical formed after decarboxylation. This prompted us to investigate the stability of **DiKTa** and **Mes_3_DiKTa** under similar conditions (see Supporting Information for details). Unfortunately, **4CzIPN**, **DiKTa**, and **Mes_3_DiKTa** all show a blue‐shift in their absorption spectra after irradiation in the presence of **1 a**, which suggests **DiKTa** and **Mes_3_DiKTa** are also unstable under these conditions; however, the products of this decomposition were not identified.

**Table 1:** Decarboxylative photo‐Giese reaction between carboxylic acids and diethyl maleate.

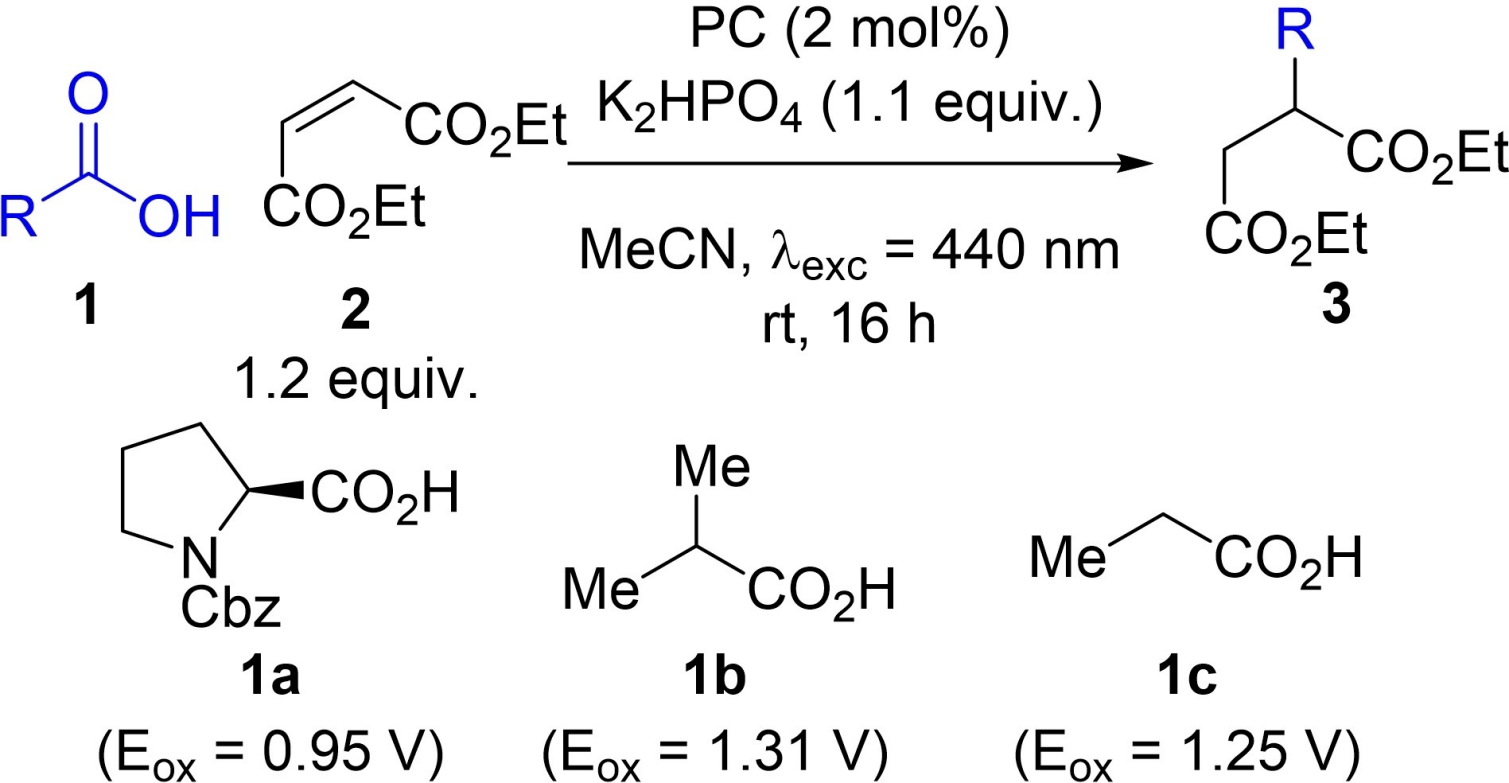			
Entry^[a]^	Acid	PC	Yield [%]^[b]^
1	1a	**DiKTa**	93 (±3)
2	1a	**Mes3DiKTa**	91 (±1)
3	1a	**4CzIPN**	95 (±4)
4	1b	**DiKTa**	78 (±3)
5	1b	**Mes_3_DiKTa**	79 (±4)
6	1b	**4CzIPN**	64 (±1)
7	1c	**DiKTa**	37 (±1)
8	1c	**Mes_3_DiKTa**	22 (±3)
9	1c	**4CzIPN**	9 (±1)

[a] Carboxylic acid (1) (0.15 mmol), diethyl maleate (2) (0.18 mmol), K_2_HPO_4_ (0.17 mmol), PC (2 mol%), MeCN (3 mL), irradiation with 440 nm LEDs, rt. [b] Yield determined by ^1^H NMR, using 1,3,5‐trimethoxybenzene as internal standard, averaged over two separate experiments.

PC loading was next investigated as a discriminating parameter. The reaction using *N*‐Cbz‐proline **1 a** as starting material was therefore repeated at 1 mol%, 0.5 mol%, 0.25 mol% and 0.1 mol% (Table 2). Yields remained largely the same for all three PCs down to 0.5 mol% (Table 2, entries 1–6). Contrastingly, differences in NMR yield were observed at 0.25 mol%, with **4CzIPN** only achieving an average yield of 28 % (Table 2, entry 7), while **DiKTa** and **Mes_3_DiKTa** maintained an average yield of 80 % (Table 2, entries 8 and 9). When using 0.1 mol% PC loading, the use of both **4CzIPN** and **DiKTa** produced poor average yields of 9 % and 18 %, respectively (Table 2, entries 10 and 11), while **Mes_3_DiKTa** achieved a significantly higher yield of 59 % (Table 2, entry 12). The evidence suggests that **DiKTa** and **Mes_3_DiKTa** perform better than **4CzIPN** at lower catalyst loadings, which is consistent with the higher molar absorptivity of **DiKTa** and **Mes_3_DiKTa** relative to **4CzIPN** at the excitation wavelength used.

**Table 2:** Catalyst loading variation of photo‐Giese reaction.

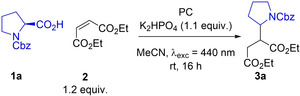			
Entry^[a]^	Catalyst Loading [mol%]	PC	Yield [%]^[b]^
1	1	**4CzIPN**	90 (±1)
2	1	**DiKTa**	88 (±2)
3	1	**Mes_3_DiKTa**	91 (±1)
4	0.5	**4CzIPN**	89 (±3)
5	0.5	**DiKTa**	78 (±4)
6	0.5	**Mes_3_DiKTa**	89 (±1)
7	0.25	**4CzIPN**	28 (±5)
8	0.25	**DiKTa**	80 (±2)
9	0.25	**Mes_3_DiKTa**	80 (±4)
10	0.1	**4CzIPN**	9 (±3)
11	0.1	**DiKTa**	18 (±5)
12	0.1	**Mes_3_DiKTa**	59 (±7)

[a] Cbz‐Pro‐H (**1 a**) (0.15 mmol), diethyl maleate (**2**) (0.18 mmol), K_2_HPO_4_ (0.17 mmol), PC, MeCN (3 mL), irradiation with 440 nm LEDs, rt. [b] Yield determined by ^1^H NMR, using 1,3,5‐trimethoxybenzene as internal standard, averaged over three separate experiments.

### Oxidative quench

Subsequent studies assessed these PCs in an oxidative quenching process based upon the atom transfer radical addition (ATRA) reaction developed by Pirtsch et al.^[20]^ Using perfluorobutyl iodide **4 a** (*E*_p_^red^=−1.42 V vs. SCE in MeCN)^[21]^ and *tert*‐butyl‐*N*‐allyl carbamate **5** as the substrates, **4CzIPN** produced the desired ATRA product **6 a** in 83 % yield (Table 3, entry 1). Interestingly, when using **DiKTa** and **Mes_3_DiKTa**, near quantitative yields of 97 % and 93 %, respectively, could be achieved (Table 3, entries 2–3). Phenacyl bromide **4 b** and diethyl bromomalonate **4 c** were then chosen as additional substrates with reduction potentials of *E*_p_^red^=−1.21 V vs. SCE and *E*_p_^red^ of −1.41 V vs. SCE in DMF, respectively (Figures S5 and S7). When using phenacyl bromide **4 b**, **4CzIPN**, **DiKTa** and **Mes_3_DiKTa** performed similarly, generating **6 b** in 71 %, 77 % and 76 % yields, respectively (Table 3, entry 4–6). However, when using **4 c**, **4CzIPN** gave marginally improved product yields, affording **6 c** in 76 % compared to 69 % and 71 % for **DiKTa** and **Mes_3_DiKTa**, respectively, although the difference between them is within the error observed for this reaction (Table 3, entries 7–9). The reduction potential stated for **4 b** by Pirtsch et al. of *E*_p_^red^=−0.49 V vs. SCE was originally reported by Tanner et al.^[22]^ and is commonly used in the literature; however, this value is erroneous and occurs only as a result of electrochemical degradation (Figure S6). Indeed, a previous report by Bahamonde et al.^[23]^ found that the peak reduction potential is significantly more negative E_p_^red^=−1.39 vs. SCE in MeCN, and our own measurements have found it to be E_p_^red^=−1.21 V vs. SCE in DMF from the peak of the differential pulse voltammogram (DPV). Similarly, literature values for the reduction potential of **4 c** vary significantly from E_p_^red^=−1.0 V vs. SCE^[24]^ in DMF to E_p_^red^=−1.74 vs. SCE^[23]^ in MeCN; our own measurements indicate that E_p_^red^=−1.41 V vs. SCE in DMF from the peak of the DPV. Such a negative reduction potential would be predicted to be beyond the ability of any of these photocatalysts to reduce via oxidative quenching; however, quenching experiments with both **4CzIPN** and **DiKTa** (see Figures S8–S11) show quenching to occur in the presence of **4 c** in DMF, showing that while endergonic, oxidative quenching does occur.

**Table 3:** Oxidative quench ATRA reaction between alkyl halides and alkene.

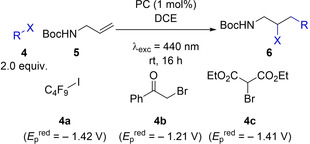			
Entry	Alkyl Halide	PC	Yield [%]^[c]^
1^[a]^	4a	**4CzIPN**	83 (±1)
2^[a]^	4a	**DiKTa**	97 (±2)
3^[a]^	4a	**Mes_3_DiKTa**	93 (±0)
4^[b]^	4b	**4CzIPN**	71 (±4)
5^[b]^	4b	**DiKTa**	77 (±1)
6^[b]^	4b	**Mes_3_DiKTa**	76 (±2)
7^[b]^	4c	**4CzIPN**	76 (±4)
8^[b]^	4c	**DiKTa**	69 (±3)
9^[b]^	4c	**Mes_3_DiKTa**	71 (±1)

[a] Alkyl halide (**4**) (0.60 mmol), *tert*‐butyl *N*‐allylcarbamate (**5**) (0.30 mmol), PC (1 mol%), DCE (1.5 mL), irradiation with 440 nm LEDs, rt, 24 h. [b] Alkyl halide (**4)** (0.50 mmol), *tert*‐butyl *N*‐allylcarbamate (**5**) (0.25 mmol), PC (1 mol%), DMF/H_2_O (1 : 2) (0.6 mL), irradiation with 440 nm LEDs, rt. [c] Yield determined by ^1^H NMR, using 1,3,5‐trimethoxybenzene as internal standard, averaged over two separate experiments.

### Photoinduced energy transfer (PEnT)

Having shown that **DiKTa** and **Mes_3_DiKTa** are capable photoredox catalysts, attention turned to their application in PEnT. One of the simplest examples of PEnT processes is the (*E*)/(*Z*) isomerization of alkenes. The isomerization of (*E*)‐stilbene **7 a** was used effectively by Lu et al. to compare various D−A TADF fluorophores and then correlate the results with their triplet energies (E_T_) relative to that of (*E*)‐stilbene (E_T_=2.2 eV) and (*Z*)‐stilbene (E_T_=2.5 eV).[^25^, ^26^] The crucial determinant for the efficiency of Dexter PEnT reactions is the degree of spectral overlap between the emission of the PC and the spin‐forbidden absorption of the substrate.^[2]^ A cross‐comparison of the triplet energies of the reactant and PC are typically used as a crude handle to assess whether the reaction is likely to proceed. Thus, to maximize the yield for the isomerization of the substrate by limiting the reverse reaction, *E*_T_(Substrate)≤*E*_T_(PC)<*E*_T_(Product). Using similar reaction conditions to those of Lu et al. [Ru(bpy)_3_](PF_6_)_2_ (*E*_T_=2.13 eV)^[27]^ achieved a (*Z*)/(*E*) ratio of 94 : 6 and **4CzIPN** (*E*_T_=2.53 eV)^[25]^ performed comparably with a (*Z*)/(*E*) ratio of 92 : 8 (Table 4, entry 1 and 2). When using **DiKTa** (*E*_T_=2.61 eV)^[15]^ as the photocatalyst a (*Z*)/(*E*) ratio of only 59 : 41 was observed (Table 4, entry 3), consistent with the high triplet energy of this PC, although a (*Z*)/(*E*) ratio of 61 : 39 was achieved with **Mes_3_DiKTa** (*E*_T_=2.49 eV),^[15]^ despite having a similar triplet energy to that of **4CzIPN** (Table 4, entry 4). Lu et al. observed similar off trend examples in their study, revealing the limitations of using *E*_T_ alone to evaluate the efficiency of Dexter PEnT photocatalysts. While not as effective as **4CzIPN**, these initial reactions did show that **DiKTa** and **Mes_3_DiKTa** can be used as PEnT photocatalysts. Di*iso*propyl fumarate **7 b** (*E*_T_=2.7 eV)^[25]^ was used by Lu et al. as a more challenging substrate due to its higher *E*_T_. [Ir(dF(CF_3_)ppy)_2_(dtbbpy)]PF_6_ (*E*_T_=2.67 eV)^[27]^ was used as the reference PC for this reaction and achieved a (*Z*)/(*E*) ratio of 95 : 5 (Table 4, entry 5). Matching with previous reports, **4CzIPN** only achieved trace amounts of isomerization, giving a (*Z*)/(*E*) ratio of 4 : 96 (Table 4, entry 6). Pleasingly, **DiKTa** gave a (*Z*)/(*E*) ratio of 90 : 10, which is comparable to that of the iridium PC (Table 4, entry 7). **Mes_3_DiKTa** afforded a lower (*Z*)/(*E*) ratio of 58 : 42 (Table 4, entry 8). Due to its higher *E*_T_, **DiKTa** should be considered as a complementary PC to **4CzIPN**, capable of engaging in more energetically demanding PEnT reactions.

**Table 4:** (*E*)/(*Z*) Isomerization of alkenes.

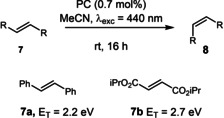				
Entry^[a]^	Alkene	PC	*E*_T_ [eV]	(*Z*)/(*E*)^[b]^
1	7a	[Ru(bpy)_3_](PF_6_)_2_	2.13	94 : 6 (±0)
2	7a	**4CzIPN**	2.53	92 : 8 (±0)
3	7a	**DiKTa**	2.62	59 : 41 (±1)
4	7a	**Mes_3_DiKTa**	2.49	61 : 39 (±2)
5	7b	[Ir(dF(CF_3_)ppy)_2_(dtbbpy)]PF_6_	2.67	95 : 5 (±1)
6	7b	**4CzIPN**	2.53	6 : 94 (±1)
7	7b	**DiKTa**	2.62	90 : 10 (±0)
8	7b	**Mes_3_DiKTa**	2.49	57 : 43 (±0)

[a] Alkene (**7**) (0.60 mmol), PC (0.7 mol%), MeCN (3 mL), irradiation with 440 nm LEDs, rt. [b] Determined using ^1^H NMR, averaged over two separate experiments.

### Dual Photoredox Catalysis

Metallaphotoredox catalysis is a fast growing area of research as it often offers a mild alternative to existing transition metal catalytic reactions and give access to different redox couples of the co‐catalyst, resulting in new reactivity.^[28]^ Nickel in particular has been paired with photocatalysts for a wide range of different coupling reactions with Luo and Zhang^[12]^ reporting the first use of **4CzIPN** as a photocatalyst in a dual‐mode catalysed (C)sp^3^−(C)sp^2^ cross‐coupling. Employing a modified version of this reaction to assess the performance of **DiKTa** and **Mes_3_DiKTa**, using **4CzIPN**, the coupling reaction between **1 a** and aryl bromide **9** gave the desired product **10** in 78 % yield (Table 5, entry 1). Both **DiKTa** and **Mes_3_DiKTa** gave similar results of 78 % and 72 %, respectively (Table 5, entries 2 and 3), providing further evidence of the versatility of these two PCs.

**Table 5:** Dual catalysed (C)sp^3^−(C)sp^2^ cross‐coupling reaction.

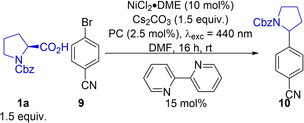		
Entry^[a]^	PC	Yield [%]^[b]^
1	**4CzIPN**	78 (±2)
2	**DiKTa**	78 (±2)
3	M**es_3_DiKTa**	72 (±1)

[a] Cbz‐Pro‐H (**1 a**) (0.225 mmol), 4‐bromobenzonitrile (**9**) (0.15 mmol), NiCl_2_⋅DME (10 mol%), Cs_2_CO_3_ (0.225 mmol), 2,2’‐bipyridine (15 mol%) PC (2.5 mol%), DMF (3.5 mL), irradiation with 440 nm LEDs, rt. [b] Yield determined by ^1^H NMR, using 1,3,5‐trimethoxybenzene as internal standard, averaged over two separate experiments.

Hydrogen atom transfer (HAT) catalysts are also commonly partnered with photocatalysts and have been used for dehalogenation reactions. A recent example, reported by Constantin et al.,^[29]^ used the alkyl radicals generated after the reductive quenching between PC* and triethylamine to abstract iodine atoms from alkyl iodides **11** to generate alkyl radicals that typically would require a far more potent reductant (E_red_(R−I)<−2 V). These alkyl radicals can then be trapped by a thiol HAT catalyst to generate the dehalogenation products **12** (Table 6). Under the literature conditions using **4CzIPN**, an 85 % yield of **12** was obtained (Table 6, entry 1). Both **DiKTa** and **Mes_3_DiKTa** were able to achieve comparable average yields of 88 % and 86 %, respectively (Table 6, entries 2 and 3).

**Table 6:** Dual catalysed deiodination.

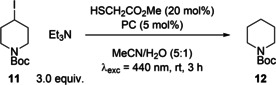		
Entry^[a]^	PC	Yield [%]^[b]^
1	**4CzIPN**	85 (±0)
2	**DiKTa**	88 (±3)
3	**Mes_3_DiKTa**	86 (±6)

[a] Alkyl iodide (**11**) (0.15 mmol), triethylamine (0.45 mmol), methyl 2‐mercaptoacetate (20 mol%), PC (5 mol%), MeCN/H_2_O (5 : 1) (1.5 mL), irradiation with 440 nm LEDs, rt. [b] Yield determined by ^1^H NMR, using 1,3,5‐trimethoxybenzene as internal standard, averaged over two separate experiments.

### Reaction profile and rates analysis

**Figure 2:**
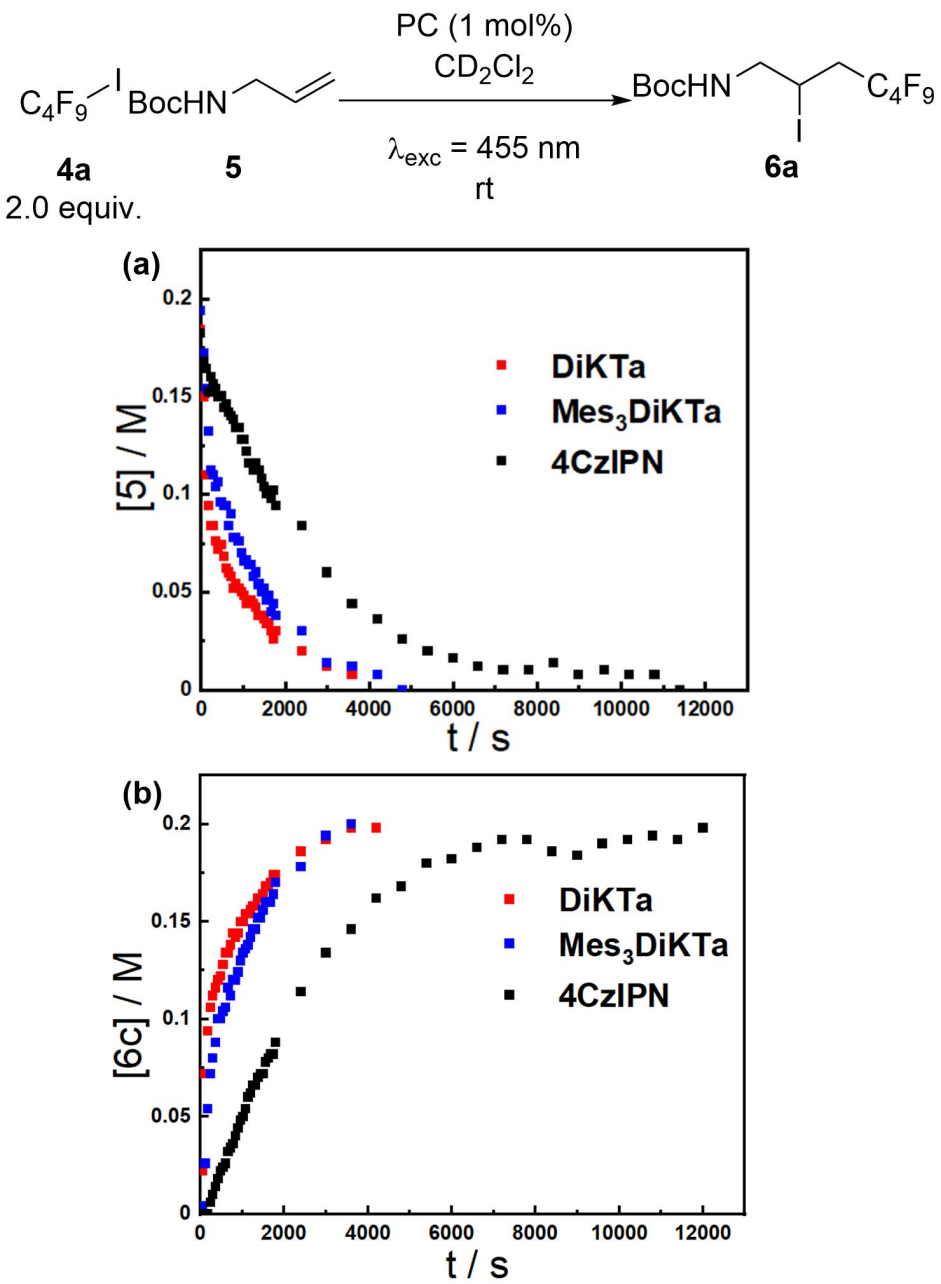
Reaction progress kinetics for the reaction in Table 3, replacing DCE with CD_2_Cl_2_ as solvent using 455 nm LED and 1,4‐bis(trimethylsilyl)benzene as internal standard. (a) Concentration of **5** over time. (b) Concentration of **6 a** over time.

Reactions in the previous sections have used the yield of the reaction after a given time to compare the efficiency of the PCs. While useful, this provides an incomplete picture of these reaction processes as it does not allow comparison of relative rates of product formation. This prompted an investigation into the reaction kinetics of the PCs for a model transformation, with the ATRA reaction between **4 a** and **5** shown in Table 3 chosen. Inspired by Yi et al.^[30]^ in situ NMR was used to monitor the generation of product employing each of the PCs (Figure 2). Notably, using this experimental set‐up, the reaction reached completion for all three PCs in less than 3 h, significantly faster than using a photoreactor, presumably due to more efficient irradiation within the in situ NMR set‐up. Furthermore, when catalysed by **Mes_3_DiKTa** or **DiKTa** the rate of product formation is significantly enhanced than with **4CzIPN**. To quantify these differences, initial rates of the reaction with each photocatalyst were calculated and compared, with the use of **DiKTa** giving a slightly larger initial rate (5×10^−4^ m s^−1^) than **Mes_3_DiKTa** (3.7×10^−4^ m s^−1^), but with both an order of magnitude larger than that of **4CzIPN** (0.6×10^−4^ m s^−1^) (Table 7).

**Table 7:** Initial rates of ATRA between **5** and **6 c**.

Entry	PC	Initial Rates [10^4^ m s^−1^]^[a]^
1	**4CzIPN**	0.6 (±0.1)
2	**DiKTa**	5 (±1)
3	**Mes_3_DiKTa**	3.7 (±0.3)

[a] Rates to 50 % conversion over two separate runs.

While our original hypothesis was that increased molar absorptivity at the excitation wavelength for **DiKTa** and **Mes_3_DiKTa** compared to **4CzIPN** would lead to increased reaction rates at 455 nm the molar absorptivity for **DiKTa** and **4CzIPN** are similar (see Figure S2). It is not evident what is the cause for the divergence in reaction rates between the two photocatalysts.

## Conclusion

In summary this manuscript demonstrates the potential of MR‐TADF compounds as a new class of photocatalyst, using **DiKTa** and **Mes_3_DiKTa** as examples compared with **4CzIPN** as a prototypical donor‐acceptor TADF benchmark. Compared to other photocatalysts **DiKTa** stands out for its wide redox window, low molecular weight, and low cost. Multiple different classes of photocatalytic reaction were tested, including, oxidative and reductive quenching reactions, energy transfer reactions and dual catalytic reactions. Both **DiKTa** and **Mes_3_DiKTa** were shown to be comparable or superior in all examples of photoredox catalysis, particularly at low catalyst loadings, and complementary for energy transfer reactions compared to **4CzIPN** due to their higher triplet energies. In situ NMR studies were used to probe reaction kinetics of the ATRA reaction between a perfluorinated alkyl halide and an unactivated alkene and showed a significant enhancement of the rate of reaction when using either **DiKTa** or **Mes_3_DiKTa** over **4CzIPN**. To the best of our knowledge this work documents the first instance of MR‐TADF compounds being used in photocatalysis and we expect further work to expand to other MR‐TADF compounds of different structural classes.

## Experimental Section

**Decarboxylative photo‐Giese reaction**: Carboxylic acid (0.15 mmol, 1.0 equiv.), K_2_HPO_4_ (31.4 mg, 0.17 mmol, 1.1 equiv.), photocatalyst (3.0 μmol, 0.02 equiv.) and diethyl maleate (27 μl, 0.18 mmol, 1.2 equiv.) were added to a vial in the photoreactor then evacuated and backfilled with nitrogen three times. In a separate Schlenk flask acetonitrile was sparged for 10 minutes and then added to the reaction vial (3 mL). The reaction was then stirred under 440 nm irradiation at rt for 16 h. Water (5 mL) was added then the mixture was extracted with CH_2_Cl_2_ (3×5 mL). The organic phases were combined and dried (Na_2_SO_4_) then concentrated *in vacuo*. Purification by silica column chromatography EtOAc:Pet. Ether afforded the desired products.

**Oxidative quench ATRA reaction for 4 a/b**: *tert*‐Butyl allyl carbamate (39.3 mg, 0.25 mmol, 1.0 equiv.), alkyl halide (0.50 mmol, 2.0 equiv.) and the photocatalyst (2.5 μmol, 0.01 equiv.) were added to a vial in the photoreactor then evacuated and backfilled with nitrogen three times. In a separate Schlenk flask a DMF/H_2_O (1 : 2) solution was sparged for 10 minutes and then added to the reaction vial (0.6 mL). The reaction was then stirred under 440 nm irradiation at rt for 16 h. Water (5 mL) and EtOAc (5 mL) were added, and the mixture was extracted with EtOAc (3×5 mL). The organic phases were combined and dried (Na_2_SO_4_) then concentrated *in vacuo*. Purification by silica column chromatography EtOAc:Pet. Ether afforded the desired products.

**Oxidative quench ATRA reaction for 4 c**: *tert*‐Butyl allyl carbamate (47.2 mg, 0.30 mmol, 1.0 equiv.), alkyl halide (0.60 mmol, 2.0 equiv.) and the photocatalyst (3.0 μmol, 0.01 equiv.) were added to a vial in the photoreactor then evacuated and backfilled with nitrogen three times. In a separate Schlenk flask DCE was sparged for 10 minutes and then added to the reaction vial (1.5 mL). The reaction was then stirred under 440 nm irradiation at rt for 16 h. The reaction was then transferred to a round‐bottom flask and concentrated *in vacuo*. Purification by silica column chromatography EtOAc:Pet. Ether afforded the desired product.

**(*E*)/(*Z*) isomerization of alkenes**: The alkene (0.40 mmol) and the photocatalyst (2.8 μmol) were added to a vial in the photoreactor then evacuated and backfilled with nitrogen three times. In a separate Schlenk flask acetonitrile was sparged for 20 minutes and then added to the reaction vial (2 mL). The reaction was then stirred under 440 nm irradiation at rt for 16 h. The reaction mixture was then transferred to a round‐bottom flask and concentrated *in vacuo*. Purification by silica column chromatography EtOAc : Pet. Ether afforded the desired products.

**Dual catalysed (C)*sp*** ^**3**^ **−(C)*sp*** ^**2**^ **Cross Coupling Reaction**: 4‐Bromobenzonitrile (27.3 mg, 0.15 mmol, 1.0 equiv.), *N*‐Cbz‐L‐proline (56.1 mg, 0.225 mmol, 1.5 equiv.), Cs_2_CO_3_ (73.3 mg, 0.225 mmol, 1.5 equiv.), 2,2’‐bipyridine (3.5 mg, 0.0225 mmol, 0.15 equiv.), NiCl_2_ ⋅ DME (3.3 mg, 0.015 mmol, 0.10 equiv.) and the photocatalyst (3.75 μmol, 0.01 equiv.) were all added to a vial in the photoreactor and evacuated then backfilled with nitrogen three times. In a separate Schlenk flask DMF was sparged for 10 minutes with nitrogen and then added to the reaction vial (3.5 mL). The reaction was then stirred under 440 nm irradiation at rt for 16 h. Water (5 mL) and EtOAc (5 mL) were added, and the mixture was extracted with EtOAc (3×5 mL). The organic phases were combined and dried (Na_2_SO_4_) then concentrated *in vacuo*. Purification by silica column chromatography EtOAc : Pet. Ether afforded the desired product.

**Dual catalysed deiodination**: 4‐Iodo‐*N*‐*boc*‐piperidine (46.7 mg, 0.15 mmol, 1.0 equiv.) and the photocatalyst (7.50 μmol, 0.05 equiv.) were added to a vial in the photoreactor and evacuated then backfilled with nitrogen three times. In a separate Schlenk flask a solution of triethylamine (62.7 μl, 0.45 mmol, 3.0 equiv.), acetonitrile (1.25 mL) and water (0.25 mL) was degassed via three freeze‐pump‐thaw cycles then transferred to the vial in the photoreactor. In a separate vial methyl thioglycolate (2.7 μl, 30 μmol. 0.2 equiv.) was sparged with nitrogen for 5 minutes before being added to the vial in the photoreactor. The reaction was then stirred under 440 nm irradiation at rt for 4 h. Then 0.5 mL of a stock solution of 1,3,5‐trimethoxybenzene in acetonitrile (0.1 m) was added and the solution stirred for 5 minutes. A sample was taken and concentrated in vacuo then dissolved in CDCl_3_ to obtain an NMR yield. A reference sample was prepared for comparison from piperidine using a known literature procedure.^[31]^

## Conflict of interest

The authors declare no conflict of interest.

1

## Supporting information

As a service to our authors and readers, this journal provides supporting information supplied by the authors. Such materials are peer reviewed and may be re‐organized for online delivery, but are not copy‐edited or typeset. Technical support issues arising from supporting information (other than missing files) should be addressed to the authors.

Supporting Information

## Data Availability

The data that support the findings of this study are openly available in Pure at https://doi.org/10.17630/52579ba6‐7023‐436f‐8242‐e89df725dca9, reference number 281110021.
