# Supplementary material for: Multi‐Resonant Thermally Activated Delayed Fluorescent (MR‐TADF) Compounds as Photocatalysts
Source: Chemistry. 2022 Nov 14;29(2):e202202998. doi: 10.1002/chem.202202998 (PMC10099988; doi:10.1002/chem.202202998)
Supplement: Supplementary file 1 — Supporting Information [file CHEM-29-0-s001.pdf]

Chemistry–A European Journal

Supporting Information

Multi-Resonant Thermally Activated Delayed Fluorescent (MR-TADF) Compounds as Photocatalysts

Callum Prentice, James Morrison, Andrew D. Smith,* and Eli Zysman-Colman*

Table of Contents

Experimental Section S2
Photocatalysis S4
Absorption Spectroscopy S9
Solvatochromism S10
Cost Comparison of **DiKTa** and **4CzIPN** S12
Cyclic Voltammetry S14
Stern-Volmer Quenching Experiments S17
In-Situ NMR Experiments S22
Photodecomposition Experiments S25
Compound Characterization S29
GCMS Traces S34
NMR Spectra S41

Experimental Section

General Synthetic Procedures. The following starting materials were synthesised according to literature materials, Cbz-Pro-H,[1] tert-butyl 4-iodopiperidine-1-carboxylate[2] and diisopropyl fumarate,[3] all other reagents and solvents were obtained from commercial sources and used as received. Photocatalysts $[Ru(bpy)_3](PF_6)_2$,[4] $[Ir(dF(CF_3)ppy)_2(dtbbpy)](PF_6)$,[5] 4CzIPN,[6] DiKTa,[7] and Mes_3DiKTa[7] were synthesised according to literature materials. Flash column chromatography was carried out using silica gel (Silia-P from Silicycle, 60 Å, 40-63 μm). Analytical thin-layer-chromatography (TLC) was performed with silica plates with aluminum backings (250 μm with F-254 indicator). TLC visualization was accomplished by 254/365 nm UV lamp. GCMS analysis was conducted using a Shimadzu QP2010SE GC-MS equipped with a Shimadzu SH-Rtx-1 column (30 m × 0.25 mm). ^{1}H, ^{13}C and ^{19}F NMR spectra were recorded on a Bruker Advance spectrometer (500 MHz for ^{1}H, 125 MHz for ^{13}C, 471 MHz for ^{19}F and 202 MHz for ^{31}P). The following abbreviations have been used for multiplicity assignments: "s" for singlet, "d" for doublet, "t" for triplet, "q" for quartet, "br" for broad, "m" for multiplet. ^{1}H and ^{13}C NMR spectra were referenced residual solvent peaks with respect to TMS (δ = 0 ppm). Melting points were measured using open-ended capillaries on an Electrothermal 1101D Mel-Temp apparatus and are uncorrected. High-resolution mass spectrometry (HRMS) was performed by SIRCAMS at University of Edinburgh.

Photophysical measurements. Optically dilute solutions of concentrations on the order of 10^{-5} or 10^{-6} M of the photocatalysts were prepared in spectroscopic or HPLC grade solvents for absorption and emission analysis. Absorption spectra were recorded at room temperature on a Shimadzu UV-2600 double beam spectrophotometer with a 1 cm quartz cuvette. Molar absorptivity determination was verified by linear regression analysis of values obtained from five independent solutions at varying concentrations with absorbance ranging from 5.11×10^{-5} to 1.18×10^{-5} M. Steady-state emission, excitation spectra and time-resolved emission spectra were recorded at 298 K using an Edinburgh Instruments F980 or a Perkin Elmer LS55 spectrofluorometer, equipped with a Hamamatsu R928 phototube. Samples were excited at 400 nm for steady-state measurements and time-resolved

measurements. Fitting of time-resolved luminescence measurements: Time-resolved PL measurements were fitted to a sum of exponentials decay model, with chi-squared ($\chi 2$) values between 1 and 2, using the EI FLS980 or Edinburgh FLS920 software. Each component of the decay is assigned a weight, (wi), which is the contribution of the emission from each component to the total emission.

Electrochemistry measurements. Cyclic Voltammetry (CV) analysis was performed on an Electrochemical Analyzer potentiostat model 620E from CH Instruments at a sweep rate of 100 mV/s. Samples were prepared as *N*,*N*-dimethylformamide (DMF) solutions, which were degassed by sparging with solvent-saturated argon gas for 5 minutes prior to measurements. All measurements were performed using 0.1 M solution of tetra-*n*-butylammonium hexafluorophosphate ([nBu$_4$N]PF$_6$]). An Ag/Ag$^+$ electrode was used as the reference electrode while a glassy carbon electrode and a platinum wire were used as the working electrode and counter electrode, respectively. The redox potentials are reported relative to a saturated calomel electrode (SCE) with a ferrocenium/ferrocene (Fc/Fc$^+$) redox couple as the internal standard (0.45 V vs SCE for DMF).[8]

Photocatalysis

Photocatalysis experiments were conducted using Asynt's LightSyn Illumin8 parallel photoreactor, as shown in Figure **S1**, allowing for up to 8 parallel photoreactions (8 mL) at a time. The reactor is placed upon a magnetic stirrer plate allowing for reactions to be run with stirring. Each of the eight reaction vials are irradiated with a separate 440nm 10W LED COB chip. Room temperature is maintained with an in-built fan.

After the photoreactions were completed, the products were analysed by ^{1}H NMR spectroscopy with an internal standard, either 1,3,5-trimethoxybenzene or 1,4-(bis(trimethylsilyl)benzene). All yields shown represent the mean yield from at least two reactions with the associated standard deviation.

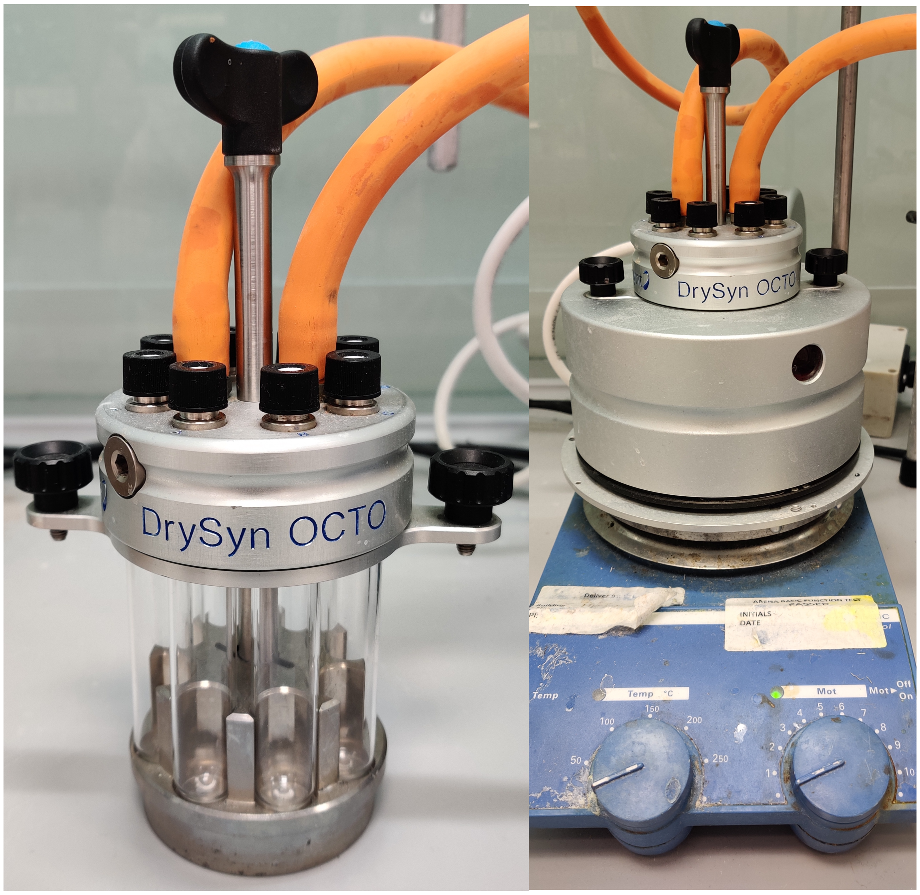

Figure **S1**. Asynt's LightSyn Illumin8 photoreactor. Left: DrySyn OCTO unit. Right: Full set-up for photochemical reactions.

Decarboxylative Photo-Giese Reaction

Variation of carboxylic acid

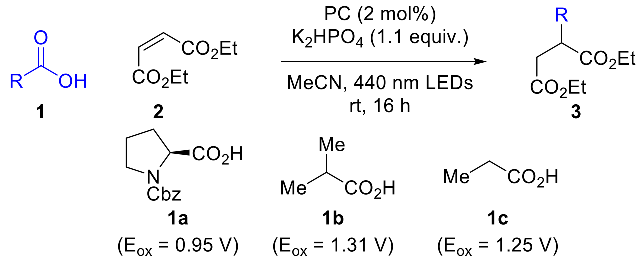

General Procedure A.

Carboxylic acid (0.15 mmol, 1.0 equiv.), K_2HPO_4 (31.4 mg, 0.17 mmol, 1.1 equiv.), photocatalyst (3.0 µmol, 0.02 equiv.) and diethyl maleate (27 µl, 0.18 mmol, 1.2 equiv.) were added to a vial in the photoreactor then evacuated and backfilled with nitrogen three times. In a separate Schlenk flask acetonitrile was sparged for 10 minutes and then added to the reaction vial (3 mL). The reaction was then stirred under 440 nm irradiation at rt for 16 hours. Water (5 mL) was added then the mixture was extracted with CH_2Cl_2 (3 × 5 mL). The organic phases were combined and dried (Na_2SO_4) then concentrated *in vacuo*. Purification by silica column chromatography EtOAc:Pet. Ether afforded the desired products.

Catalyst Loading Variation

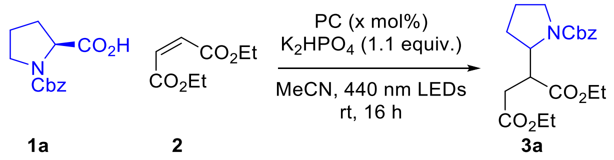

General Procedure B.

A stock solution of the photocatalyst (0.5 mM) was prepared in acetonitrile and diluted to the appropriate concentrations for each catalyst loading (1 mol%, 0.5 mol%, 0.25 mol% and 0.1 mol%). The diluted solution was then added to a Schlenk flask and degassed *via* three freeze-pump-thaw cycles. ((Benzyloxy)carbonyl)proline (37.4 mg, 0.15 mmol, 1.0 equiv.), K_2HPO_4 (31.4 mg, 0.17 mmol, 1.1 equiv.) and diethyl maleate (27 µl, 0.18 mmol, 1.2 equiv.) were added to three vials in the photoreactor then evacuated and backfilled with nitrogen three times. The photocatalyst solution was added (3 mL) and the solution stirred under 440 nm irradiation at rt for 16 hours. Then 0.5 mL of a stock solution of 1,3,5-trimethoxybenzene in acetonitrile (0.1 M) was added and the solution stirred for 5 minutes. A sample was taken and concentrated *in vacuo* then dissolved in $CDCl_3$ to obtain an NMR yield.

Oxidative Quench ATRA Reaction

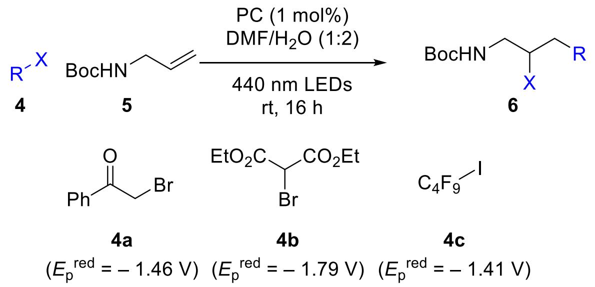

General Procedure C.

tert-Butyl allyl carbamate (39.3 mg, 0.25 mmol, 1.0 equiv.), alkyl halide (0.50 mmol, 2.0 equiv.) and the photocatalyst (2.5 µmol, 0.01 equiv.) were added to a vial in the photoreactor then evacuated and backfilled with nitrogen three times. In a separate Schlenk flask a DMF/H_2O (1:2) solution was sparged for 10 minutes and then added to the reaction vial (0.6 mL). The reaction was then stirred under 440 nm irradiation at rt for 16 hours. Water (5 mL) and EtOAc (5 mL) were added, and the mixture was extracted with EtOAc (3 × 5 mL). The organic phases were combined and dried (Na_2SO_4) then concentrated *in vacuo*. Purification by silica column chromatography EtOAc:Pet. Ether afforded the desired products.

General Procedure D.

tert-Butyl allyl carbamate (47.2 mg, 0.30 mmol, 1.0 equiv.), alkyl halide (0.60 mmol, 2.0 equiv.) and the photocatalyst (3.0 µmol, 0.01 equiv.) were added to a vial in the photoreactor then evacuated and backfilled with nitrogen three times. In a separate Schlenk flask DCE was sparged for 10 minutes and then added to the reaction vial (1.5 mL). The reaction was then stirred under 440 nm irradiation at rt for 16 hours. The reaction was then transferred to a round-bottom flask and concentrated *in vacuo*. Purification by silica column chromatography EtOAc:Pet. Ether afforded the desired product.

Photoinduced Energy Transfer Reaction

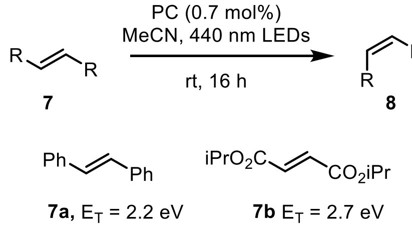

General Procedure E.

The alkene (0.40 mmol) and the photocatalyst (2.8 µmol) were added to a vial in the photoreactor then evacuated and backfilled with nitrogen three times. In a separate Schlenk flask acetonitrile was sparged for 20 minutes and then added to the reaction vial (2 mL).

The reaction was then stirred under 440nm irradiation at rt for 16 hours. The reaction mixture was then transferred to a round-bottom flask and concentrated *in vacuo*. Purification by silica column chromatography EtOAc:Pet. Ether afforded the desired products.

Dual Catalysed (C)*sp*^{3}-(C)*sp*^{2} Cross Coupling Reaction

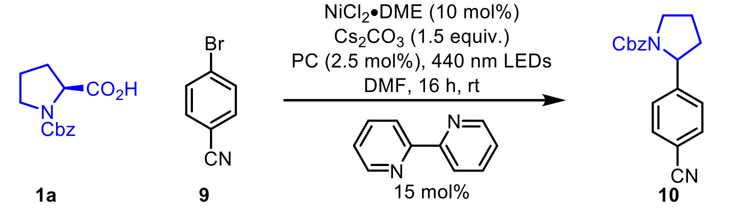

General Procedure F.

4-Bromobenzonitrile (27.3 mg, 0.15 mmol, 1.0 equiv.), *N*-Cbz-L-proline (56.1 mg, 0.225 mmol, 1.5 equiv.), Cs_2CO_3 (73.3 mg, 0.225 mmol, 1.5 equiv.), 2,2'-bipyridine (3.5 mg, 0.0225 mmol, 0.15 equiv.), $NiCl_2$•DME (3.3 mg, 0.015 mmol, 0.10 equiv.) and the photocatalyst (3.75 µmol, 0.01 equiv.) were all added to a vial in the photoreactor and evacuated then backfilled with nitrogen three times. In a separate Schlenk flask DMF was sparged for 10 minutes with nitrogen and then added to the reaction vial (3.5 mL). The reaction was then stirred under 440nm irradiation at rt for 16 hours. Water (5 mL) and EtOAc (5 mL) were added, and the mixture was extracted with EtOAc (3 × 5 mL). The organic phases were combined and dried (Na_2SO_4) then concentrated *in vacuo*. Purification by silica column chromatography EtOAc:Pet. Ether afforded the desired product.

Dual Catalysed Deiodination

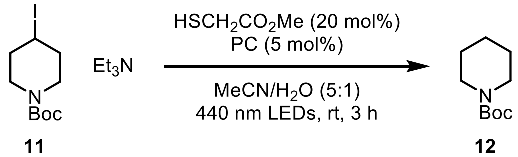

General Procedure G.

4-Iodo-*N*-*boc*-piperidine (46.7 mg, 0.15 mmol, 1.0 equiv.) and the photocatalyst (7.50 µmol, 0.05 equiv.) were added to a vial in the photoreactor and evacuated then backfilled with nitrogen three times. In a separate Schlenk flask a solution of triethylamine (62.7 µl, 0.45 mmol, 3.0 equiv.), acetonitrile (1.25 mL) and water (0.25 mL) was degassed *via* three freeze-pump-thaw cycles then transferred to the vial in the photoreactor. In a separate vial methyl thioglycolate (2.7 µl, 30 µmol. 0.2 equiv.) was sparged with nitrogen for 5 minutes before being added to the vial in the photoreactor. The reaction was then stirred under 440nm irradiation at rt for 4 hours. Then 0.5 mL of a stock solution of 1,3,5-trimethoxybenzene in acetonitrile (0.1 M) was added and the solution stirred for 5 minutes. A sample was taken and concentrated in vacuo then dissolved in $CDCl_3$ to obtain an NMR

yield. A reference sample was prepared for comparison from piperidine using a known literature procedure.[9]

Absorption Spectroscopy

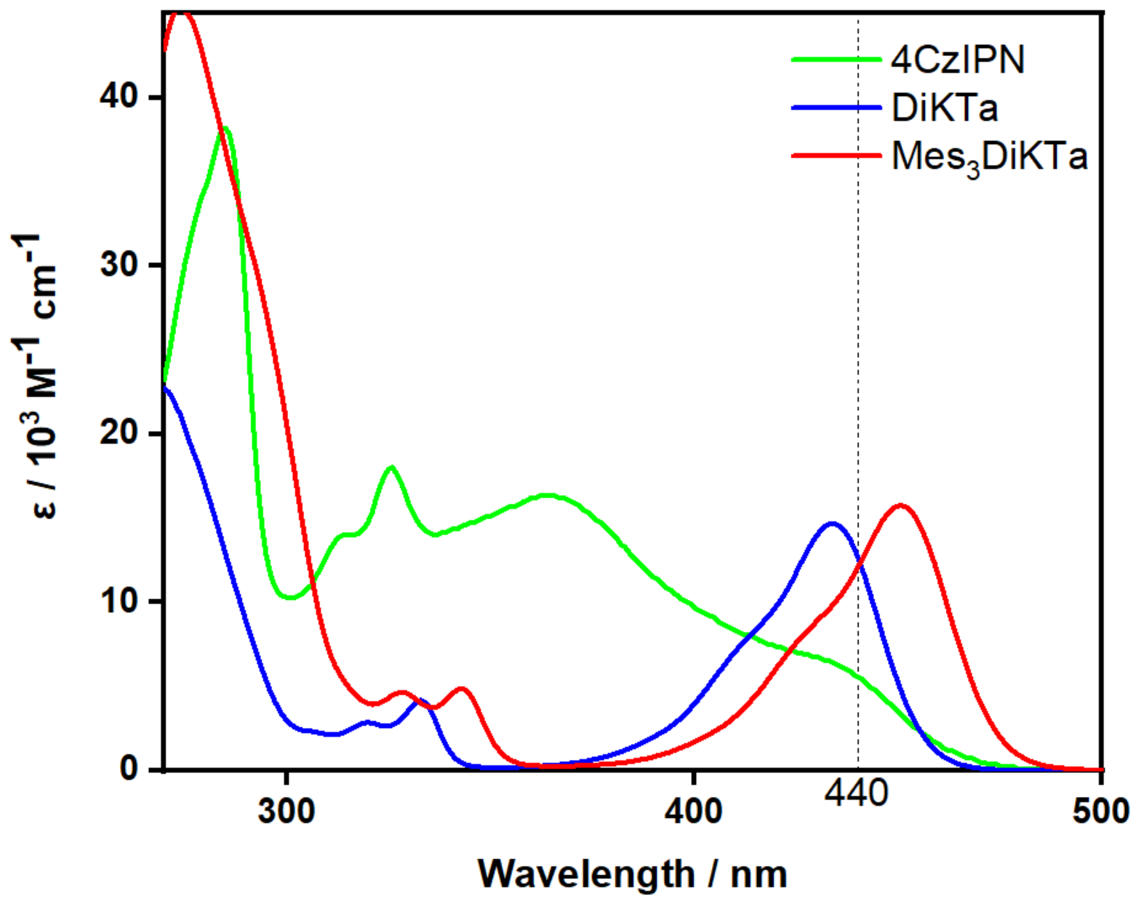

Figure S2. Absorption spectra of **4CzIPN**, **DiKTa** and **Mes$_3$DiKTa** in MeCN with wavelength of photoreactor LEDs marked as a vertical dashed line.

Solvatochromism

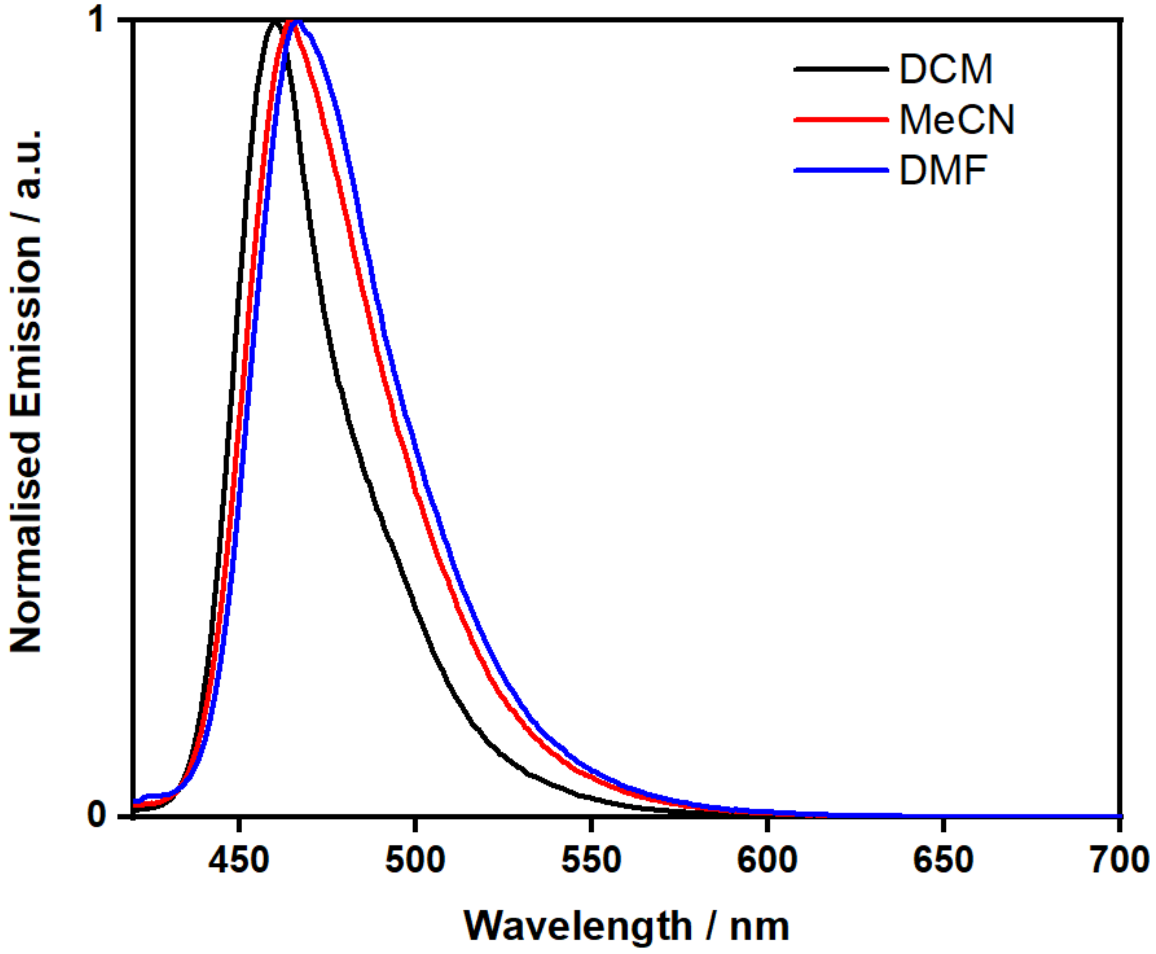

Figure **S3**. Solvatochromic study for **DiKTa**, λ_{exc} = 400 nm, measurements performed at room temperature under air.

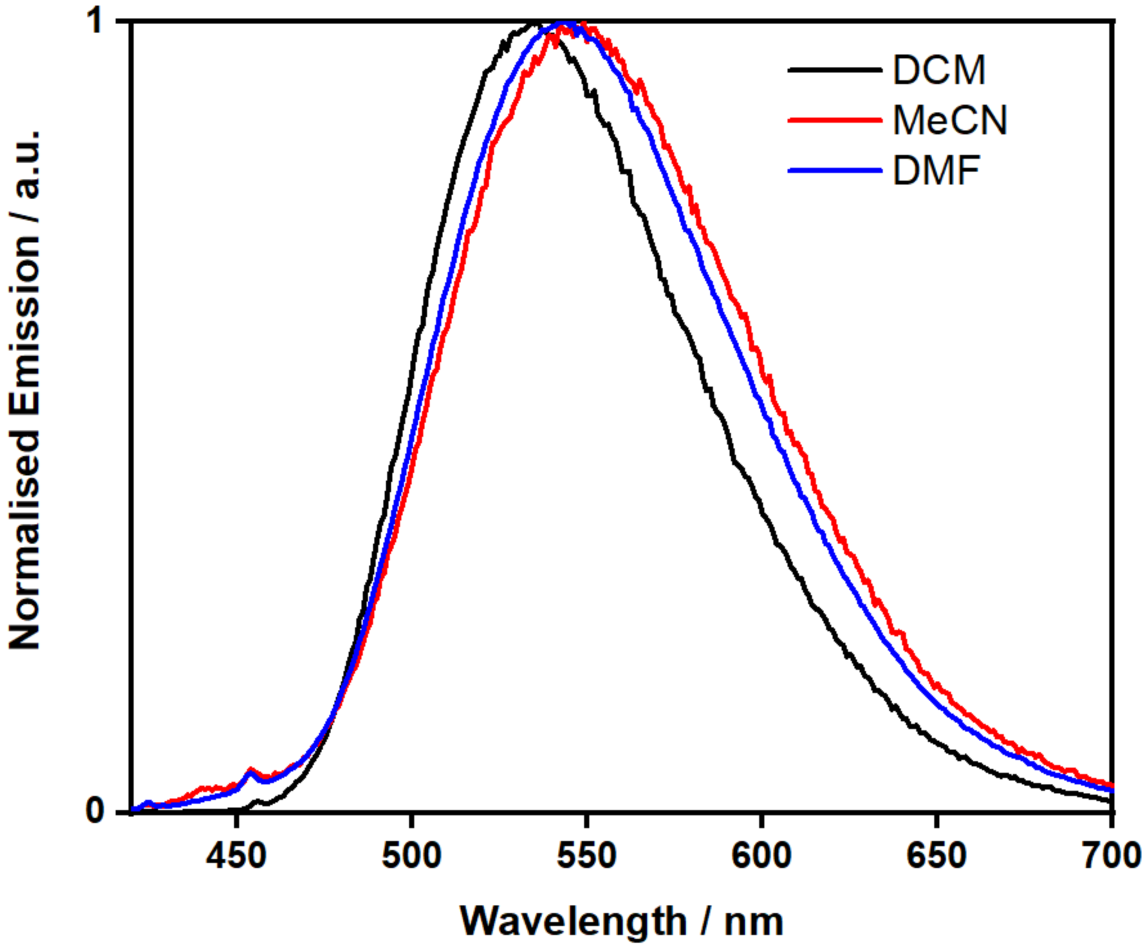

Figure **S4**. Solvatochromic study for **4CzIPN**, λ_{exc} = 400 nm, measurements performed at room temperature under air.

Cost Comparison of DiKTa and 4CzIPN

4CzIPN:

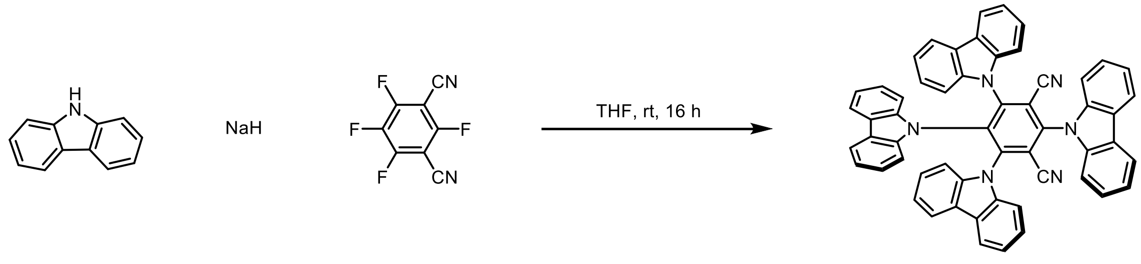

Chemical	Equivalents	Mass / g	Cost / £
Carbazole	5	1.67	0.47
NaH	7.5	0.6	0.29
Tetrafluoroisophthalonitrile	1	0.4	5.44
**4CzIPN**	**-**	1.5	6.2

Total cost/g = (0.47+0.29+5.44)/1.5 = £4.13

Total cost/mmol = (4.13*788.27)/1000 = £3.26

[a]250g bottle from Sigma. [b]100g bottle from Sigma. [c]5g bottle from Fluorochem. Prices as of 12/05/2022.

DiKTa:

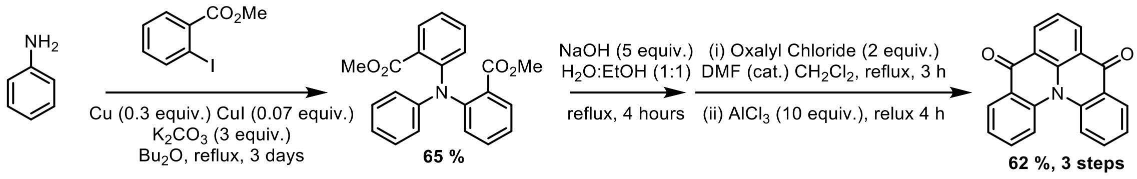

First step:

Chemical	Equivalents	Mass / g	Cost / £
Aniline	1	2.33	0.08[a]

Cu (powder)	0.3	0.48	0.07[b]
Cu(I)I	0.07	0.33	0.06[c]
K_2CO_3 (anhydrous)	3	10.4	0.31[d]
Bu_2O (anhydrous)	Solvent	19.3	2.53[e]
Methyl 2-iodobenzoate	3	19.7	3.94[f]
Diester	-	5.9	6.99

Total cost / g = (0.08+0.07+0.06+0.31+2.53+3.94)/5.9 = £1.18 / g

Total cost / mmol = (1.18*361.4)/1000 = £0.43 / mmol

[a]500g bottle from TCI Chemicals. [b]100g bottle from Alfa Aesar. [c]100g bottle from Fluorochem. [d]500g bottle from Alfa Aesar. [e]1L bottle from Sigma. [f]250g bottle from CarboSynth. Prices as of 12/05/2022.

Second Step:

Chemical	Equivalent	Mass/g	Cost/£
Diester	1	1.0	1.18[a]
NaOH	5	0.55	0.02[b]
Oxalyl Chloride	2	0.70	0.24[c]
Aluminium Chloride	10	3.69	0.18[d]
**DiKTa**	-	0.51	1.62

Total cost/g = (1.18+0.02+0.24+0.18)/0.51 = £3.18/g

Total cost/mmol = (2.31*297.31)/1000 = £0.94/mmol

[a]Calculated above. [b]500g bottle from Alfa Aesar. [c]100g bottle from TCI Chemicals. [d]500g bottle from TCI Chemicals. Prices as of 12/05/2022.

Cyclic Voltammetry

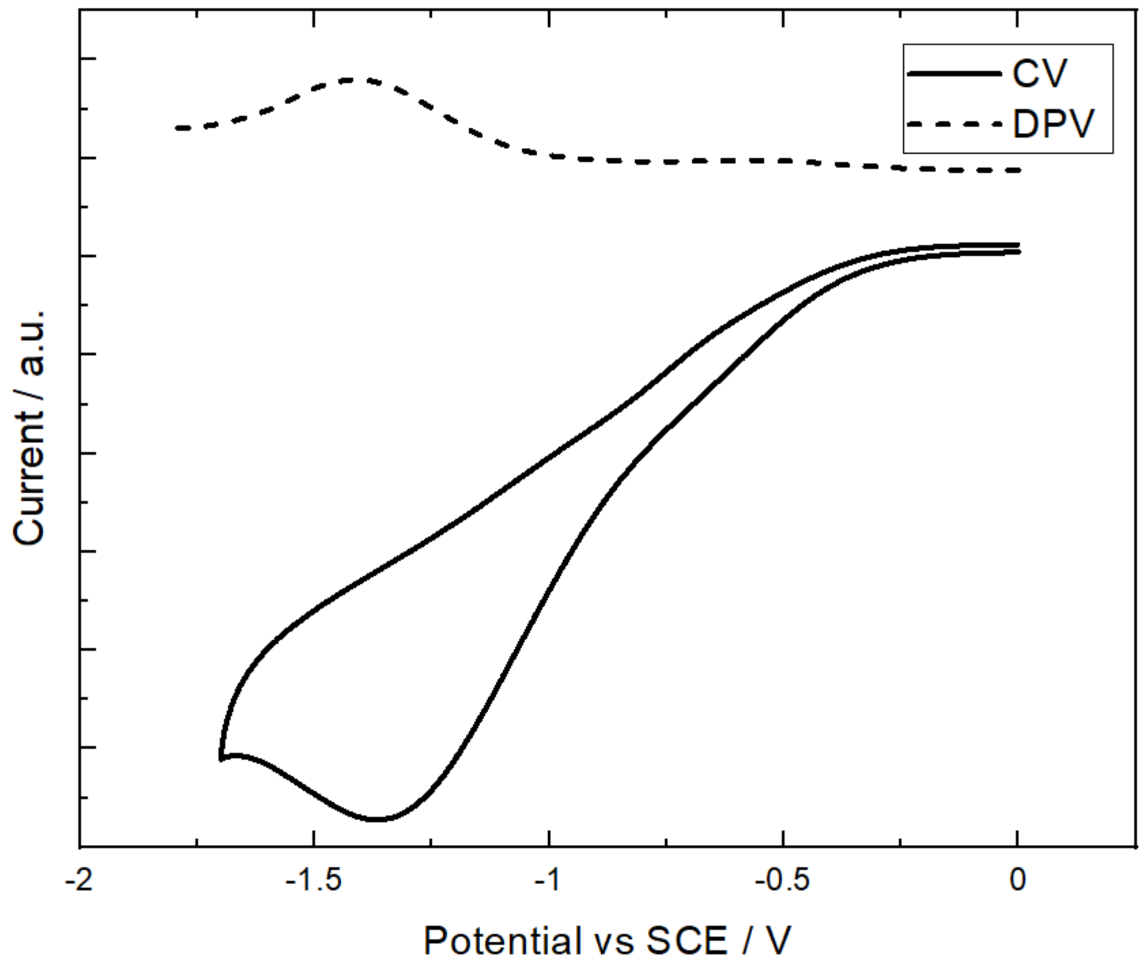

Figure **S5.** CV and DPV of diethyl bromomalonate in DMF, reported vs SCE at scan rate of 0.05 V s^{-1}.

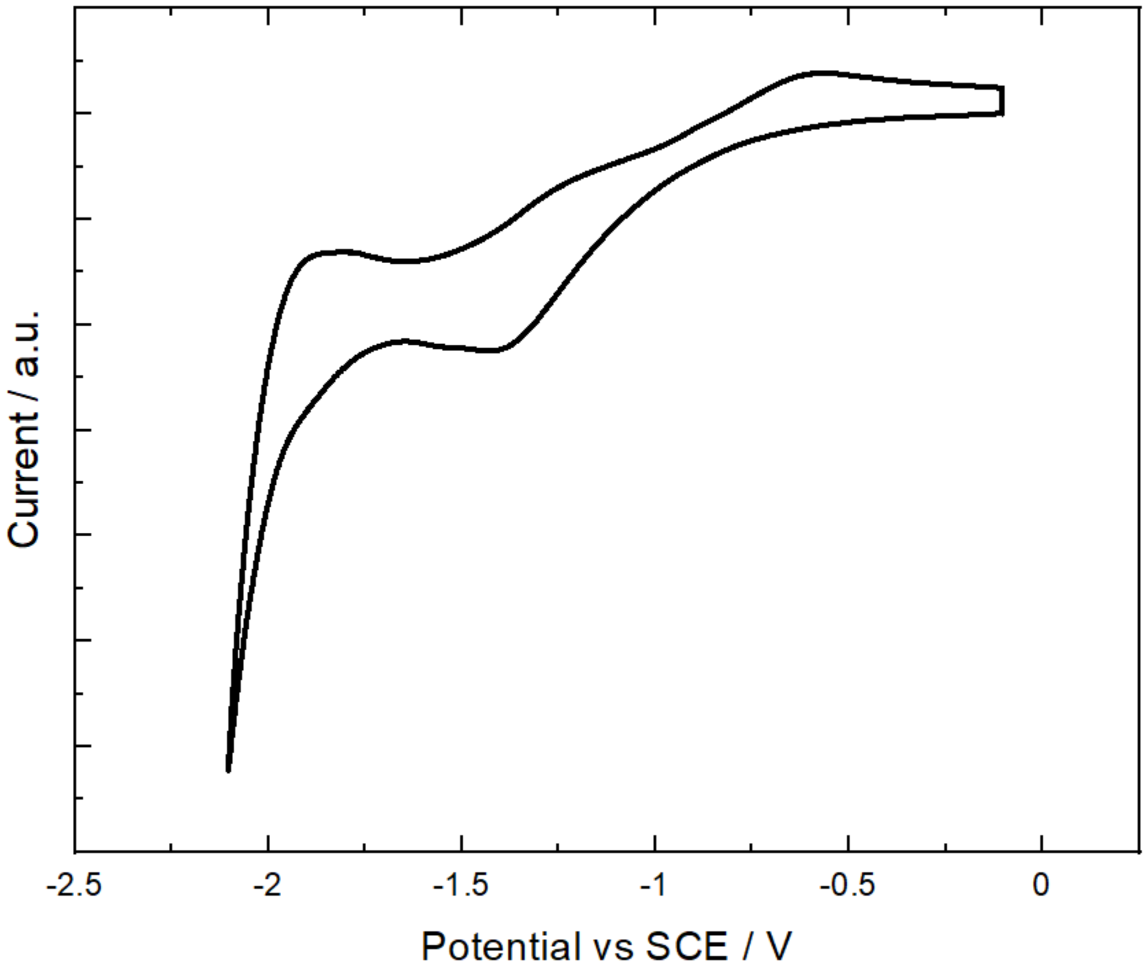

Figure **S6.** CV of phenacyl bromide in DMF, reported vs SCE at scan rate of 0.1 V s^{-1}, observed peak at – 0.6 V is due to electrochemical degradation.

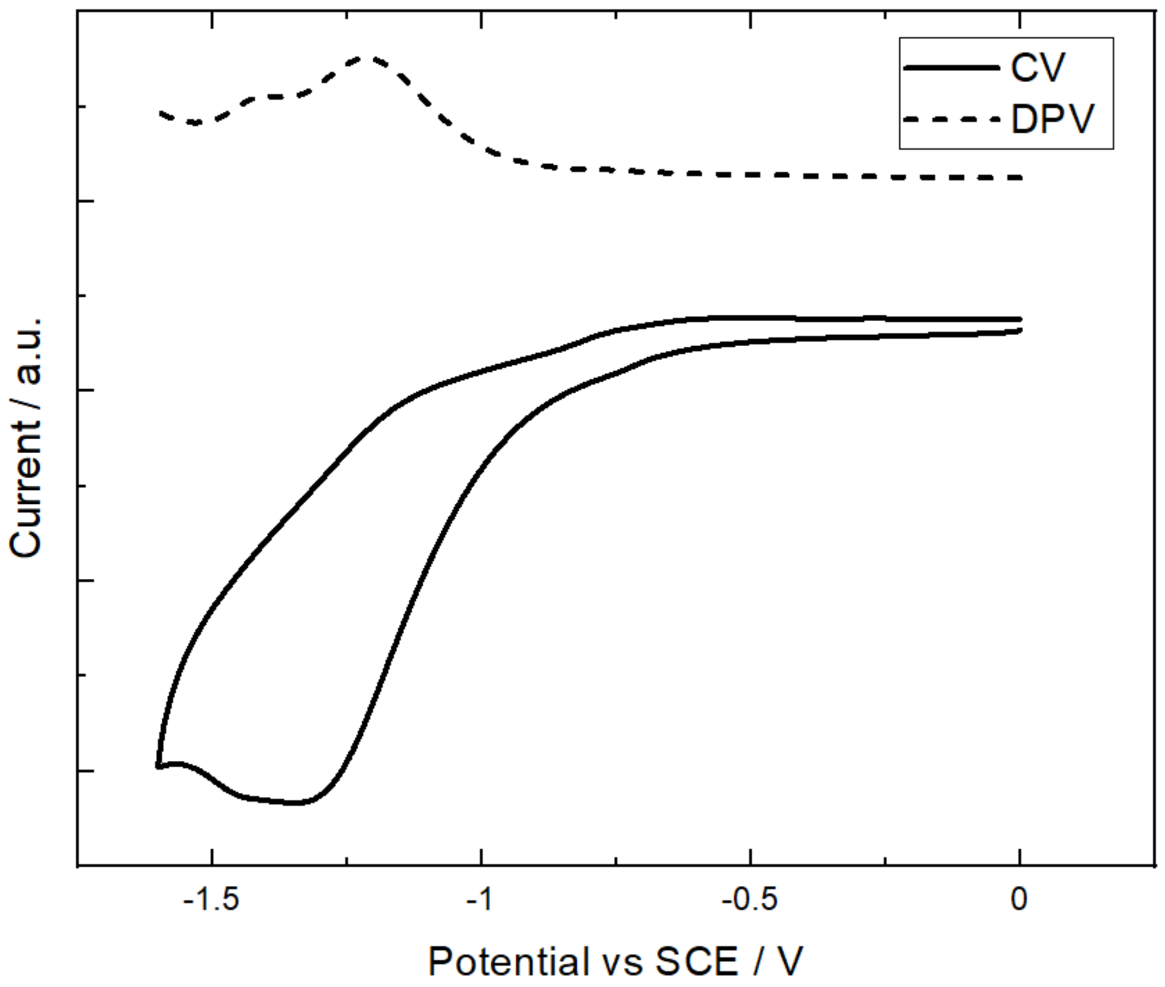

Figure **S7.** CV and DPV of phenacyl bromide in DMF, reported vs SCE at scan rate of 0.05 V s^{-1}.

Stern-Volmer Experiments

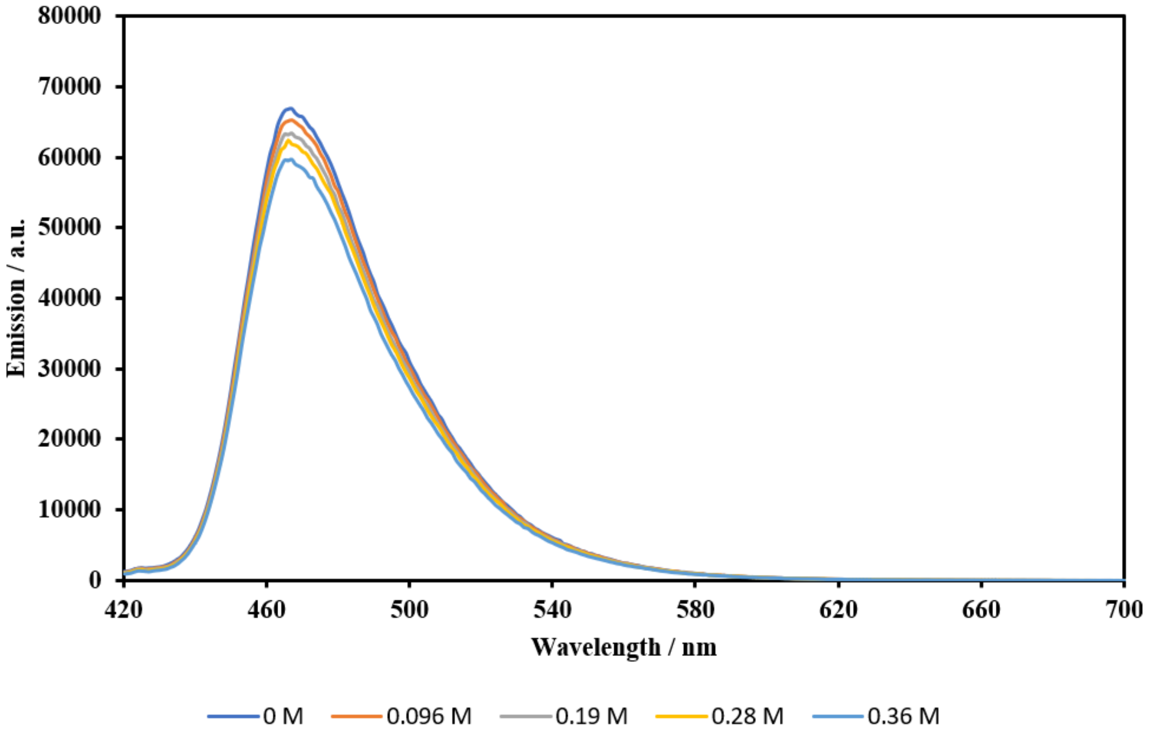

Figure **S8**. Emission quenching data of **DiKTa** by sequential addition of diethyl bromomalonate in DMF. λ_{exc} = 400 nm.

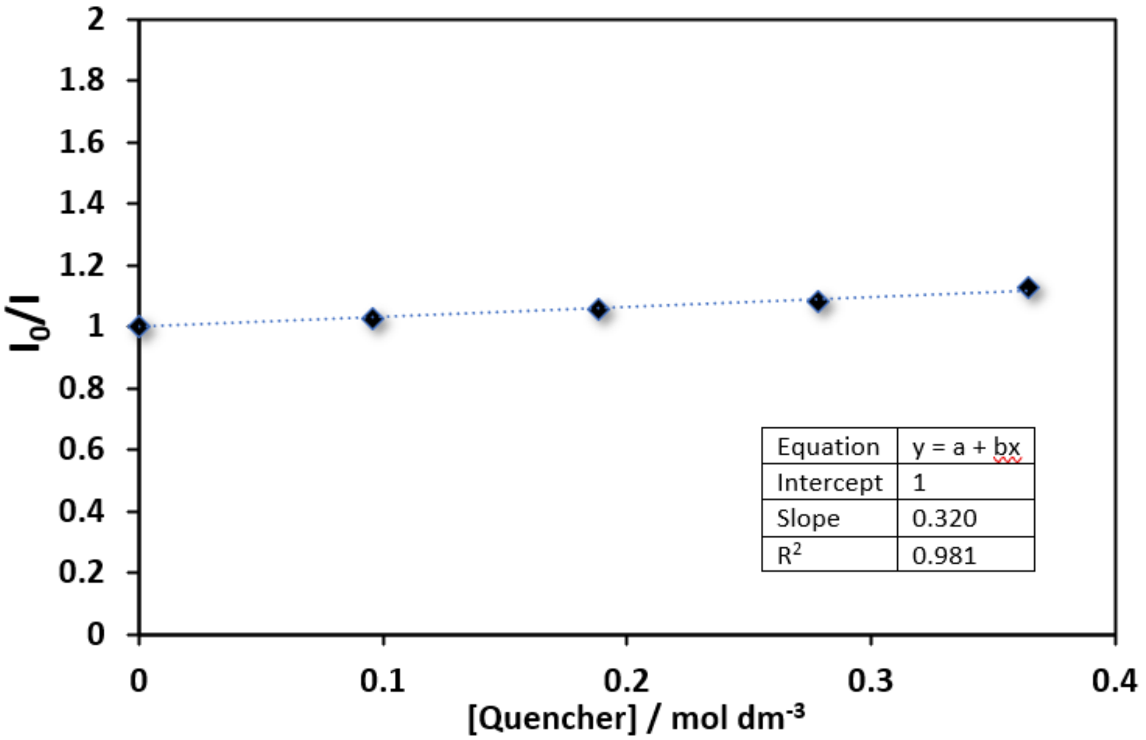

Figure **S9**. Stern-Volmer plot of the quenching of the emission of **DiKTa** in DMF by sequential addition of diethyl bromo malonate.

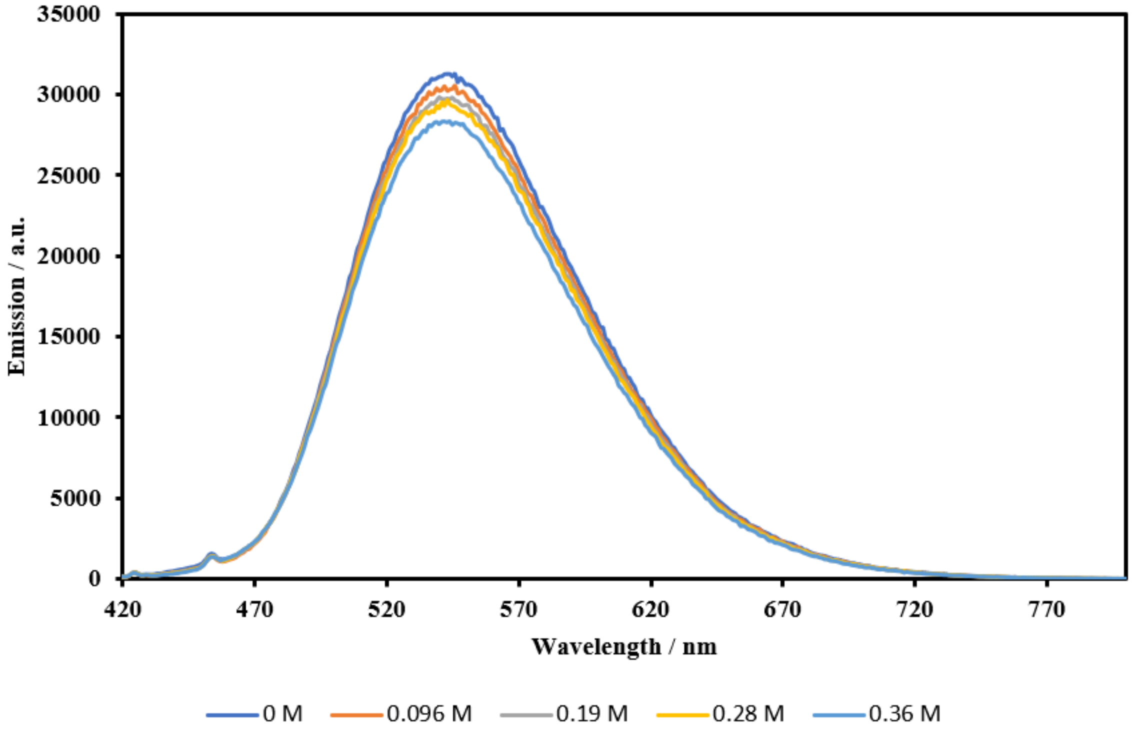

Figure **S10**. Emission quenching data of **4CzIPN** by sequential addition of diethyl bromomalonate in DMF. λ_{exc} = 400 nm.

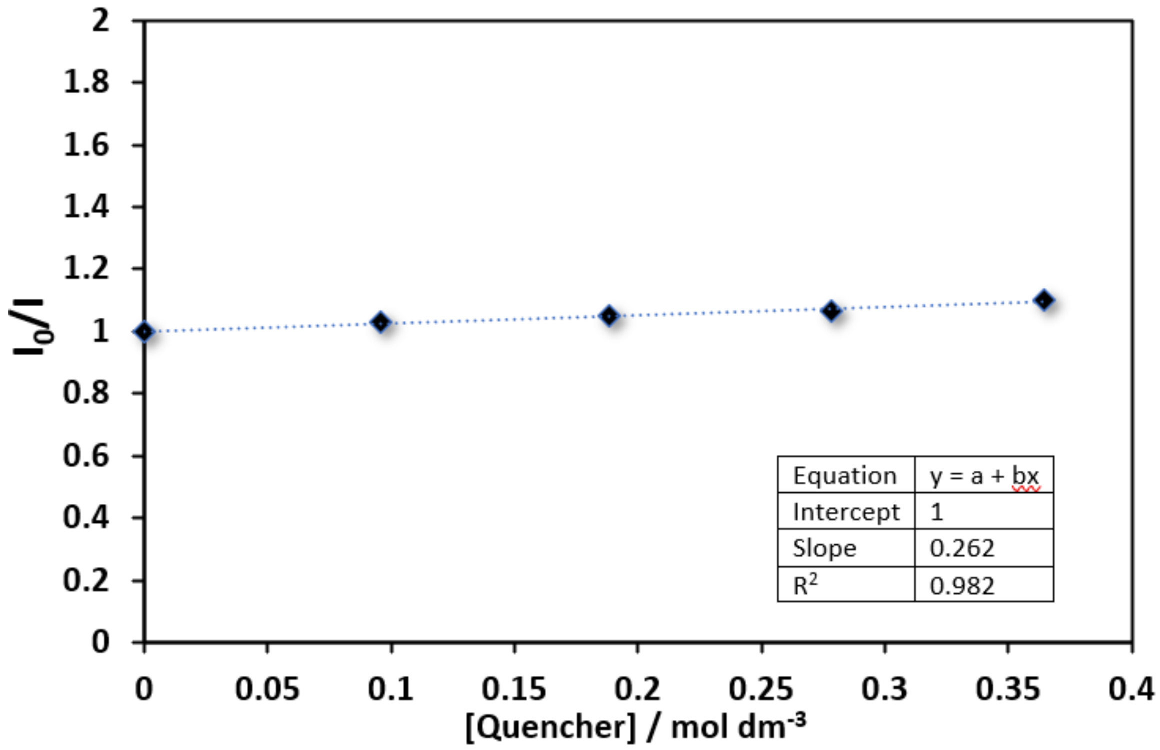

Figure **S11**. Stern-Volmer plot of the quenching of the emission of **4CzIPN** in DMF by sequential addition of diethyl bromo malonate.

Summary of Quenching Constants

DiKTa with diethyl bromomalonate:

$\tau_{PL} = 8.2 \times 10^{-9}$ s

$K_{SV} = 0.320$ mol^{-1} dm^3

$k_q = K_{SV}/\ \tau_{PL} = 3.9 \times 10^{-7}$ mol^{-1} dm^3 s^{-1}

4CzIPN with diethyl bromomalonate:

Using prompt fluorescence lifetime

$\tau_{PF} = 3.28 \times 10^{-8}$ s

$K_{SV} = 0.262$ mol^{-1} dm^3

$k_q = K_{SV}/\ \tau_{PF} = 7.98 \times 10^{-6}$ mol^{-1} dm^3 s^{-1}

Using delayed fluorescence lifetime

$\tau_{DF} = 1.91 \times 10^{-6}$ s

$K_{SV} = 0.262$ mol^{-1} dm^3

$k_q = K_{SV} / \tau_{DF} = 1.37 \times 10^{-5}$ mol^{-1} dm^3 s^{-1}

As no delayed emission is observed in DMF for **DiKTa** we have used the prompt fluorescence lifetime and expect quenching to occur from the S_1 state.

However, for **4CzIPN** delayed emission is observed in DMF so we have quoted two quenching constants. Both k_q are at least an order of magnitude larger for **DiKTa** than for **4CzIPN**, which could explain the increased rates observed in the *in-situ* NMR studies.

Kinetics Experiments

Sample Preparation:

tert-Butyl allyl carbamate (70.1 mg, 0.45 mmol, 1.0 equiv.), 1,4-bis(trimethylsilyl)benzene (25.0 mg, 0.113 mmol, 0.25 equiv.) and the photocatalyst (4.5 μmol, 0.01 equiv.) were added to a vial and sealed with a septum then evacuated and backfilled with nitrogen three times. Then nonafluoro-1-iodobutane (0.15 mL, 0.90 mmol, 2.0 equiv.) was added. In a separate Schlenk flask CD_2Cl_2 was sparged for 15 minutes with DCM saturated N_2 and then added to the vial (2.25 mL). The solution was stirred until complete dissolution then 0.45 mL transferred into an NMR tube. The coaxial insert is then quickly inserted, secured with parafilm and both wrapped in aluminium foil to block ambient light. The optical fibre is then inserted into the coaxial insert and secured in place with parafilm. Finally, the NMR loop and light source are turned on simultaneously.

Experimental Set-up:

In-situ NMR set-up is inspired by DiRocco *et al*.[10]

A - LEDD1B - T-Cube LED Driver, 1200 mA Max Drive Current – Thorlabs.

B - M455F3 - 455 nm, 17 mW (Min) Fiber-Coupled LED, 1000 mA, SMA – Thorlabs.

C - High NA Plastic Optical Fibre for in Situ LED-NMR Spectroscopy – Goldstone Scientific.

D - Z278513 - Wilmad® coaxial insert and cap – Sigma Aldrich.

E - 15318918 - Bel-Art™ SP Scienceware™ 5mm O.D. Thin Walled Precision NMR Tubes – Fisher Scientific.

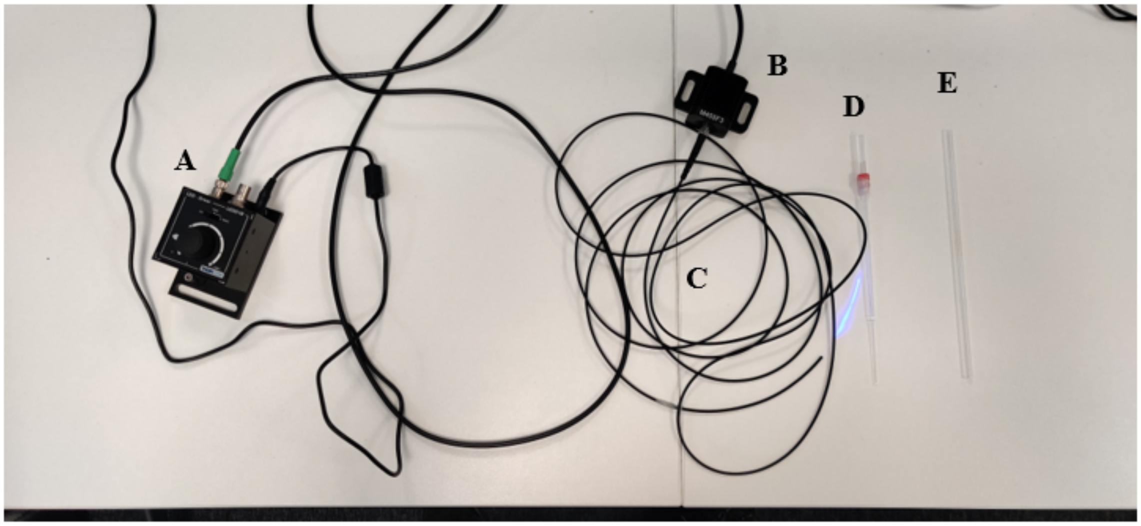

Figure **S12**. Equipment used for *in-situ* NMR experiments.

Data Collected:

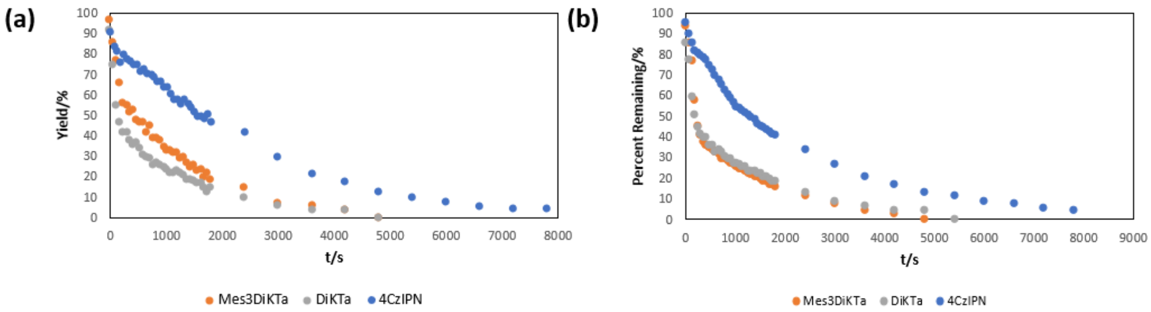

Figure **S13**. (a) Consumption of **5** over time for **4CzIPN**, **DiKTa** and **Mes$_3$DiKTa**. (b) Repeat of (b) with the same batch of each reagent.

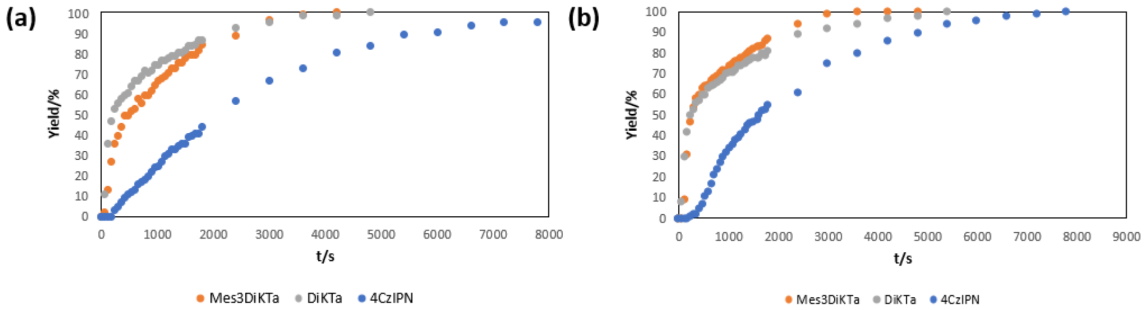

Figure **S14**. (a) Formation of **6a** over time for **4CzIPN**, **DiKTa** and **Mes$_3$DiKTa.** (b) Repeat of (a) with the same batch of each reagent.

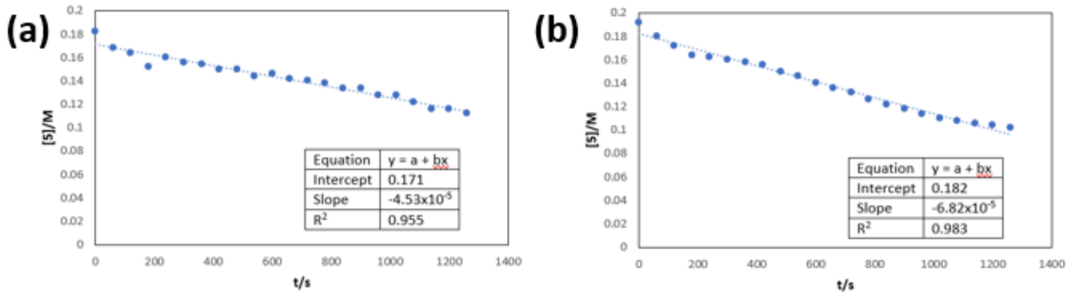

Figure **S15**. (a) Linear plot of concentration of **5** over time up to 50% conversion with **4CzIPN**. (b) Repeat of (a) with the same batch of each reagent.

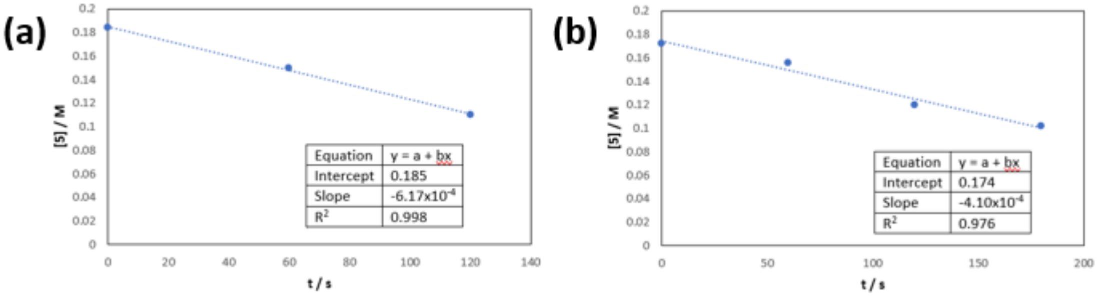

Figure **S16**. (a) Linear plot of concentration of **5** over time up to 50% conversion with **DiKTa**. (b) Repeat of (a) with the same batch of each reagent.

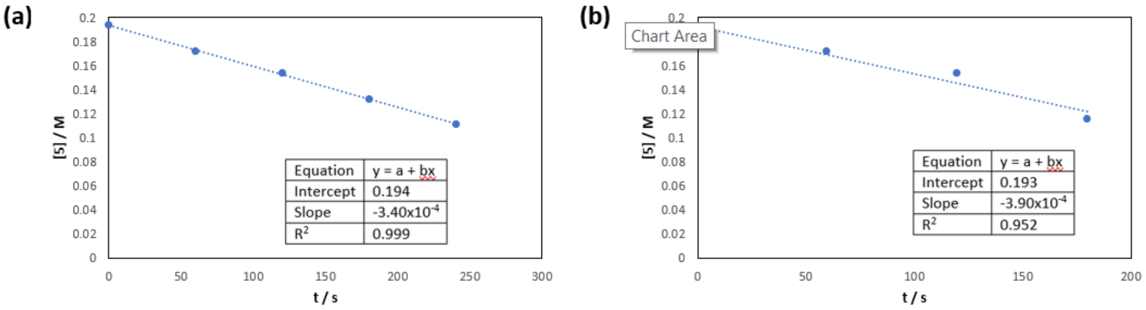

Figure **S17**. (a) Linear plot of concentration of **5** over time up to 50% conversion with **Mes_3DiKTa**. (b) Repeat of (a) with the same batch of each reagent.

Table **S1**. Initial Rates Summary

Photocatalyst	**4CzIPN**	**DiKTa**	**Mes_3DiKTa**
Initial Rates x10^4/ M s^{-1}	0.6 (±0.1)	5 (±1)	3.7 (±0.3)

Photodecomposition of the Photocatalysts

A recent study by Grotjahn and König showed that D-A phthalonitrile-containing photocatalysts such as **4CzIPN** photodecompose when irradiated in the presence of alkyl carboxylic acids *via* photosubstitution of one of the cyanide groups by an alkyl radical (Scheme **S1**).[11] Due to the loss of one of the electron-accepting groups, the monocyano product **13** possesses significantly altered optoelectronic properties, including a much larger optical gap and more negative excited state redox potentials. For example, the photosubstitution product **13** between **4CzIPN** and 2-phenylpropanoic acid **14** is a stronger photoreductant [E_{ox}(**13**$^{\bullet+}$/**13***) = – 1.43 V] but a weaker photooxidant [E_{red}(**13***/**13**$^{\bullet-}$) = 1.27 V] compared to –1.23 V/1.44 V for 4CzIPN. This increase in reducing power was exploited previously by König[12] to generate carbanions from aryl acetic acids as the active photocatalyst was in fact the photosubstitution product, rather than **4CzIPN** itself. However, photoinstability of the PC is not desirable and it could be envisaged that the decreased oxidative power of **4CzIPN** resulting from the photosubstitution could hinder the reaction. Furthermore, the observed blueshift in the absorption spectrum of the photosubstituted **4CzIPN** results in a lower molar absorptivity during the photoexcitation using a blue-light excitation source (λ_{exc} ~ 450 nm), resulting in slower reaction kinetics. Pleasingly, a much smaller blue shift and reduction of molar absorptivity is observed under the same conditions for **DiKTa** and **Mes_3DiKTa** although photodecomposition is also observed (see Figures **S17**-**S19**); the photodecomposition products were not; however, identified.

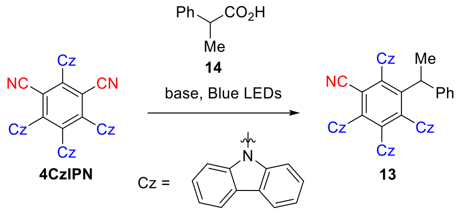

Scheme **S1.** Photosubstitution of 4CzIPN reported by König with alkyl carboxylic acids.

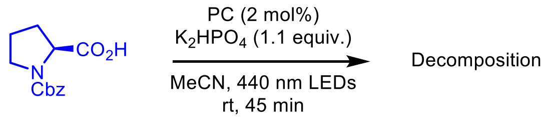

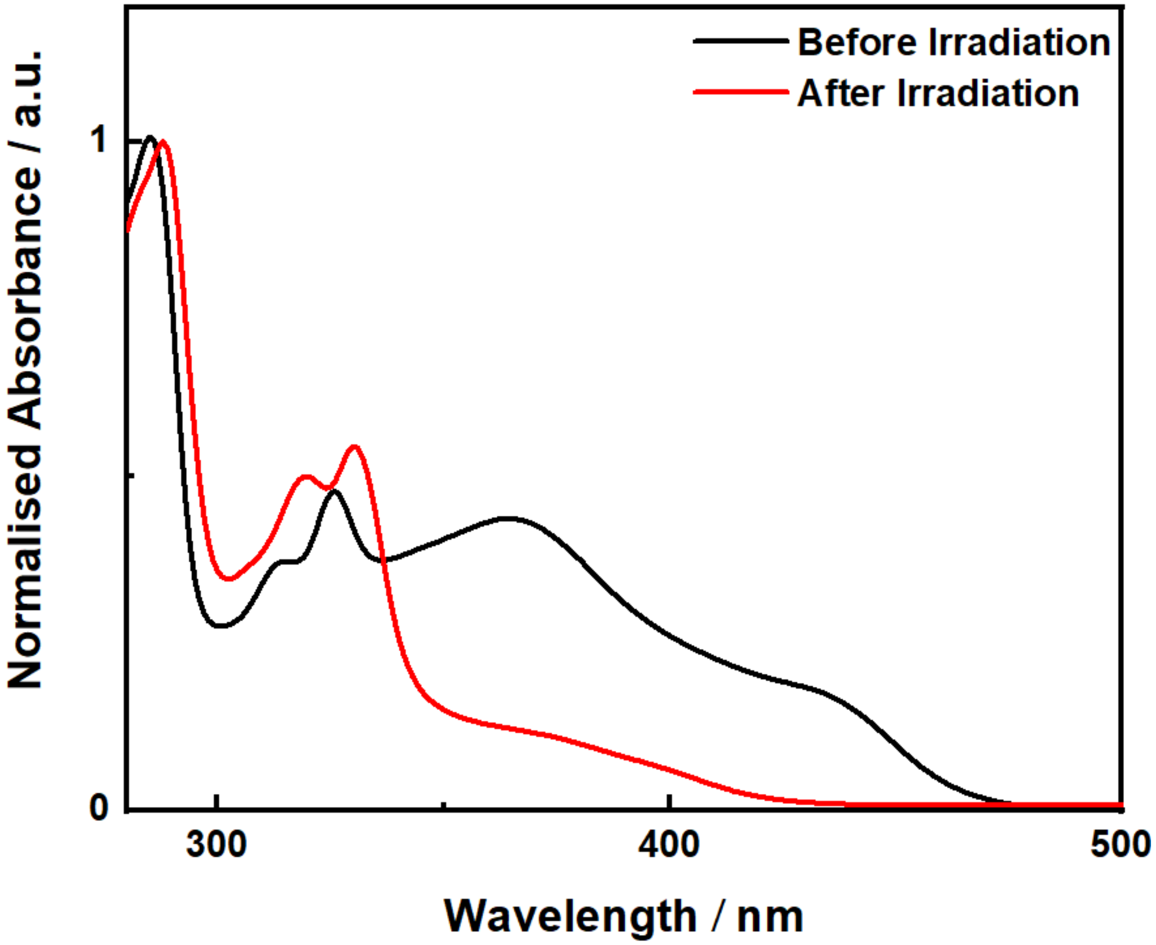

Figure **S18**. UV-Vis absorption spectra of **4CzIPN**, K_2HPO_4 and *N*-Cbz-Pro in MeCN before and after 45 minutes of irradiation under nitrogen in the photoreactor.

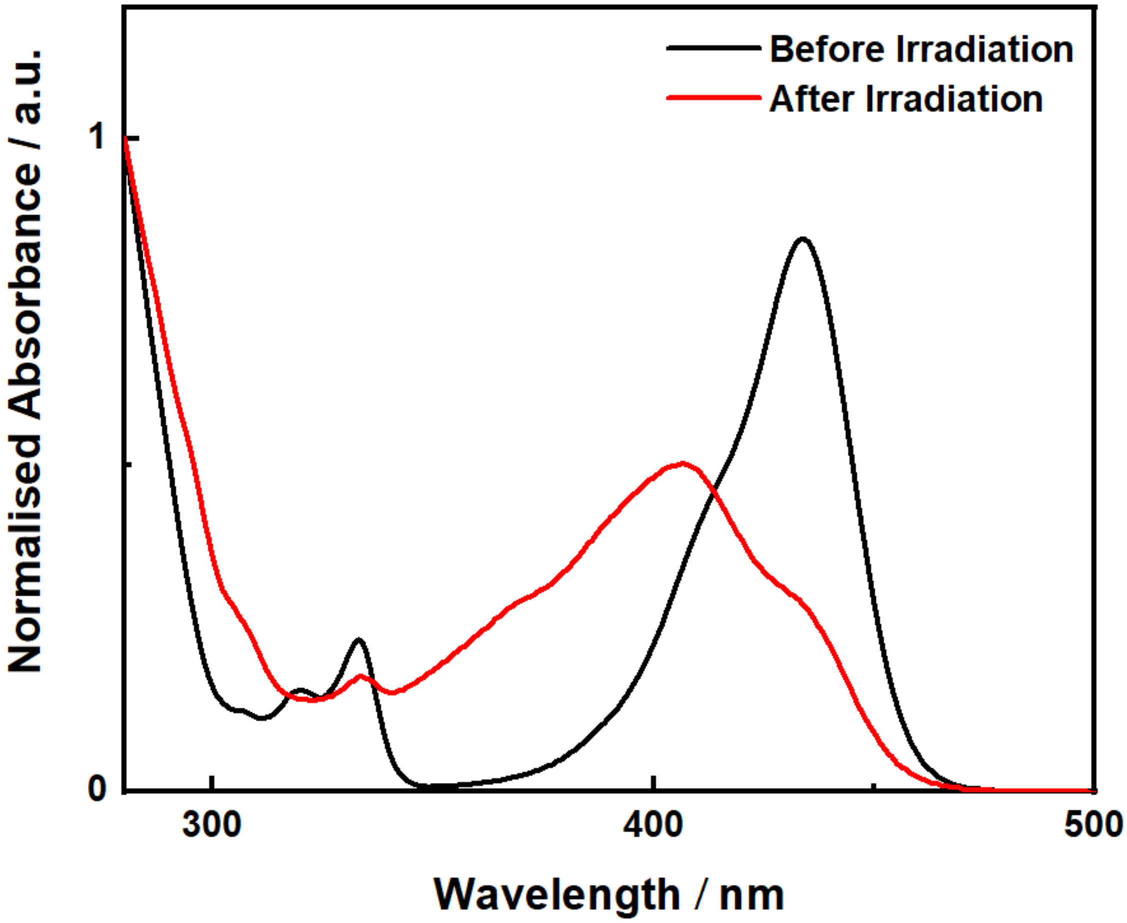

Figure **S19**. UV-Vis absorption spectra of **DiKTa**, K_2HPO_4 and *N*-Cbz-Pro in MeCN before and after 45 minutes of irradiation under nitrogen in the photoreactor.

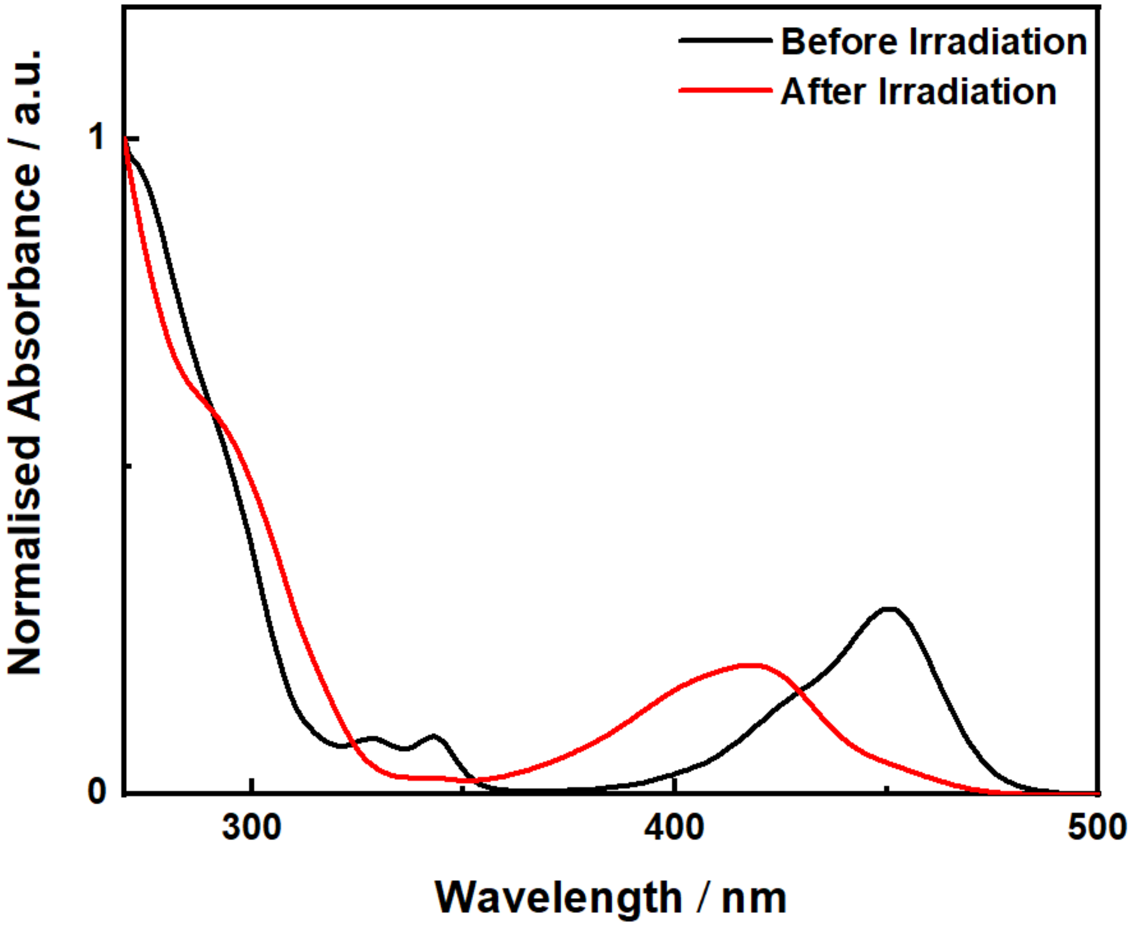

Figure **S20**. UV-Vis absorption spectra of **Mes_3DiKTa**, K_2HPO_4 and *N*-Cbz-Pro in MeCN before and after 45 minutes of irradiation under nitrogen in the photoreactor.

Compound Characterization

diethyl 2-(1-((benzyloxy)carbonyl)pyrrolidin-2-yl)succinate (3a):

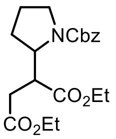

Synthesised using general procedure A.

Oil. **R*f*:** 0.2 (20% EtOAc/Pet. Ether). **^{1}H NMR (500 MHz, CDCl$_3$) δ (ppm):** 1.21 – 1.31 (6H, m, OCH$_2$C*H*$_3$), 1.75 – 2.00 (4H, m, C(2)*H*$_2$ + C(3)*H*$_2$), 2.26 – 2.55 (1H, m, C(6)*H*^{A}H^B), 2.69 – 2.86 (1H, m, C(6)H^A*H*^{B}), 3.21 – 3.36 (1H, m, C(5)*H*), 3.47 – 3.75 (2H, m, NC(1)*H*$_2$), 4.06 – 4.20 (4H, OC*H*$_2$CH$_3$), 4.31 – 4.36 (1H, m, NC(4)*H*), 5.07 – 5.27 (2H, OC*H*$_2$Ph), 7.30 – 7.45 (5H, m, Ph*H*).

GCMS $C_{11}H_{20}O_4$ [M]$^+$ found 377.3, requires 377.2, retention time = 10.5 min.

Data matches that previously reported.[13]

diethyl 2-isopropylsuccinate (3b):

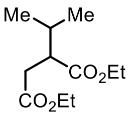

Synthesised using general procedure A.

Oil. **R*f*:** 0.15 (10% EtOAc/Pet. Ether). **^{1}H NMR (500 MHz, CDCl$_3$) δ (ppm):** 0.92 (3H, d, *J* = 6.9 Hz, (C*H*$_3$)^{A}CH(CH$_3$)^B), 0.94 (3H, d, *J* = 6.9 Hz, (CH$_3$)^{A}CH(C*H*$_3$)^B), 1.25 (6H, dt, *J* = 7.7 Hz, 7.1 Hz, OCH$_2$C*H*$_3$), 1.99 (1H, m, (CH$_3$)^{A}C*H*(CH$_3$)^B), 2.35 – 2.45 (1H, m, C*H*^{A}CH^B), 2.66 – 2.77 (2H, m, CH^{A}C*H*^{B} + CH$_2$C*H*), 4.09 – 4.18 (4H, m, OC*H*$_2$CH$_3$). **^{13}C{^{1}H} NMR (126 MHz, CDCl$_3$) δ (ppm):** 14.1 (OCH$_2$*C*H$_3$), 14.2 (OCH$_2$*C*H$_3$), 19.6 (CH(*C*H$_3$)$_2$, 20.0 (CH(*C*H$_3$)$_2$, 30.1 (*C*H(CH$_3$)$_2$), 33.2 (C(3)*C*H$_2$), 47.5 (C(2)*C*H), 60.3 (O*C*H$_2$CH$_3$), 60.5 (O*C*H$_2$CH$_3$), 172.5 (*C*OOEt), 174.3 (*C*OOEt).

Infra-Red (ν max, cm^{-1}): 2964 (C-H), 1732 (C=O).

HRMS (EI) $C_{11}H_{20}O_4$ [M]$^+$ found 216.1361, requires 216.1356 (+2.3 ppm)

diethyl 2-ethylsuccinate (3c):

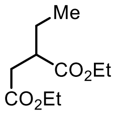

Synthesised using general procedure A.

Mixture with diethyl succinate and diethyl maleate. **R*f*:** 0.10 (10% EtOAc/Pet. Ether). **^{1}H NMR (500 MHz, CDCl$_3$) δ (ppm):** 0.92 (3H, t, *J* = 7.5 Hz, CH$_2$C*H*$_3$), 1.25 (6H, m, OCH$_2$C*H*$_3$) 1.53 – 1.71 (2H, m, C*H*$_2$CH$_3$), 2.41 (1H, dd, *J* = 15.9 Hz, 4.6 Hz, C*H*^{A}CH^B), 2.66 – 2.81 (2H, m, CH^{A}C*H*^{B} + CH$_2$C*H*), 4.09 – 4.17 (4H, m, OC*H*$_2$CH$_3$).

GCMS $C_{11}H_{20}O_4$ [M+H]$^+$ found 203.2, requires 203.1, retention time = 4.5 min.

Data matches that previously reported.[14]

tert-butyl (4,4,5,5,6,6,7,7,7-nonafluoro-2-iodoheptyl)carbamate (6a):

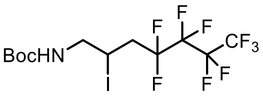

Synthesised using general procedure D.

Brown solid. **mp** 67-69 °C {Lit.[15] 68-70 °C} **R*f*:** 0.60 (40% EtOAc/Pet. Ether). **^{1}H NMR (400 MHz, CDCl$_3$) δ (ppm):** 1.46 (9H, s, OC(C*H*$_3$)$_3$), 2.69 – 2.95 (2H, m, CF$_2$C*H*$_2$), 3.41 – 3.52 (1H, m, NHC*H*^{A}CH^B), 3.54 – 3.66 (1H, m, NHCH^{A}C*H*^{B}), 4.31 – 4.43 (1H, m, CH$_2$CI*H*CH$_2$), 4.96 (1H, s, N*H*). **^{19}F NMR (376 MHz, Solvent) δ (ppm):** -125.81 – -125.95 (2F, m), -124.57 (2F q, *J* = 9.4 Hz), -113.32 (1F, t, *J* = 13.5 Hz), - 113.75 (1F, t, *J* = 13.5 Hz), -81.03 – -80.93 (3F, m).

Data matches that previously reported.[16]

tert-butyl (2-bromo-5-oxo-5-phenylpentyl)carbamate (6b):

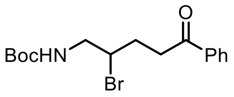

Synthesised using general procedure C.

Colourless Solid. **Mp** 112-114 °C **R_f:** 0.40 (40% EtOAc/Pet. Ether). **^{1}H NMR (400 MHz, $CDCl_3$) δ (ppm):** 1.45 (9H, s, OC(CH_3)$_3$), 2.11 – 2.29 (1H, m, C(O)C$H^A$$H^B$), 2.31 – 2.42 (1H, m, C(O)C$H^A$$H^B$) 3.10 – 3.36 (2H, m, C(O)CH_2CH_2), 3.44 – 3.57 (1H, m, NHC$H^A$$H^B$), 3.58 – 3.69 (1H, m, NHC$H^A$$H^B$), 4.17-4.36 (1H, m, NH$CH_2$C*H*Br), 5.03 (1H, s, N*H*), 7.48 (2H, td, *J* 7.6, 1.4, *m*-Ph*H*), 7.55 – 7.61 (1H, m, *p*-Ph*H*), 7.96 – 8.00 (2H, m, *o*-Ph*H*).

Data matches that previously reported.[16]

diethyl 2-(2-bromo-3-((tert-butoxycarbonyl)amino)propyl)malonate (6c):

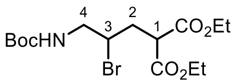

Synthesised using general procedure D.

Yellow oil. **Rf:** 0.40 (20% EtOAc/Pet. Ether). **^{1}H NMR (400 MHz, $CDCl_3$) δ (ppm):** 1.27 (6H, app. td, OCH_2CH_3, *J* = 7.1 Hz, 2.3 Hz), 1.44 (9H, C(CH_3)$_3$ s), 2.27 (1H, ddd, C(2)$H^A$$H^B$, *J* = 14.9 Hz, 10.1 Hz, 5.1 Hz), 2.47 (1H, ddd, C(2)$H^A$$H^B$, *J* = 14.9 Hz, 9.5 Hz, 3.7 Hz), 3.42 – 3.60 (2H, C(1)*H* + C(4)$H^A$$H^B$, m), 3.74 (1H, dd, C(4)$H^A$$H^B$, *J* = 9.5 Hz, 5.0 Hz), 4.12 (1H, q, C(3)*H*, *J* = 7.1 Hz), 4.17 – 4.27 (4H, OC$H_2$$CH_3$, m), 4.97 (1H, N*H*, br s). **^{13}C{^{1}H} NMR (126 MHz, $CDCl_3$) δ (ppm):** 14.0 (OCH_2CH_3), 14.1 (OCH_2CH_3), 28.3 (CCH_3), 34.6 (C(2)CH_2), 47.1 (C(3)*C*HBr), 50.2 (C(1)*C*H), 53.3 (C(4)CH_2), 61.76 (OCH_2) , 61.83 (OCH_2), 79.9 (O*C*(CH_3)$_3$), 155.6 (*C*(O)NH), 168.5 (*C*OOEt), 168.9 (*C*OOEt).

Data matches that previously reported.[16]

(*Z*)-Stilbene (8a):

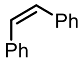

Synthesised using general procedure E.

Colourless oil. **Rf:** 0.3 (Hexane). **^{1}H NMR (400 MHz, $CDCl_3$) δ (ppm):** 6.61 (2H, s, *H*C=C*H*), 7.16 – 7.29 (10H, m, Ar*H*).

GCMS $C_{11}H_{20}O_4$ [M]$^+$ found 180.2, requires 180.1, retention time = 5.7 min.

Data matches that previously reported.[17]

Diisopropyl Fumarate (8b):

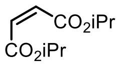

Colourless oil. **Rf:** 0.2 (20% EtOAc/Pet. Ether). **^{1}H NMR (400 MHz, $CDCl_3$) δ (ppm):** 1.29 (12H, d, CH(C*H*$_3$)$_2$, *J* = 6.3 Hz), 5.12 (2H, p, C*H*(CH$_3$)$_2$ *J* = 6.3 Hz), 6.18 (2H, s, *H*C=C*H*).

GCMS $C_{11}H_{20}O_4$ [M+H]$^+$ found 201.2, requires 200.1, retention time = 4.4 min.

Data matches that previously reported.[18]

benzyl 2-(4-cyanophenyl)pyrrolidine-1-carboxylate (10):

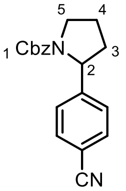

Yellow oil. **Rf:** 0.2 (30% EtOAc/Pet. Ether). **^{1}H NMR (400 MHz, $CDCl_3$) δ (ppm):** 1.78 – 1.87 (1H, m, C(4)H^AH^B), 1.87 – 1.98 (2H, m, C(3)H^AH^B + C(4)H^AH^B), 2.30 – 2.47 (1H, m, C(3)H^AH^B), 3.60 – 3.76 (2H, m, C(5)H_2), 4.81 – 4.95 (1H, m, C(2)*H*), 4.95 – 5.21 (2H, m, CO_2CH_2), 6.89 (1H, d, *J* = 7.2 Hz, Ar*H*), 7.15 – 7.25 (3H, m, Ar*H*), 7.27 – 7.40 (3H, m, Ar*H*), 7.51 – 7.63 (2H, m, Ar*H*).

GCMS $C_{11}H_{20}O_4$ [M]$^+$ found 306.3, requires 306.1, retention time = 10.7 min.

Data matches that previously reported.[19]

tert-butyl piperidine-1-carboxylate (12):

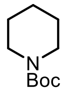

Colourless oil. **Rf:** 0.2 (10% EtOAc/Pet. Ether) **^{1}H NMR (400 MHz, $CDCl_3$) δ (ppm):** 1.45 (9H, s. $(CH_3)_3$), 1.47 – 1.59 (6H, m, $CH_2CH_2CH_2CH_2CH_2$), 3.31 – 3.41 (4 H, m, CH_2NCH_2).

GCMS $C_{11}H_{20}O_4$ [M]$^+$ found 185.2, requires 185.1, retention time = 4.4 min.

Data matches that previously reported.[9]

GCMS Data

Sample Information

Analyzed by	: Admin
Analyzed	: 22/05/2022 15:19:44
Sample Type	: Unknown
Level #	: 1
Sample Name	: proline addition repurify
Sample ID	:
IS Amount	: [1]=1
Sample Amount	: 1
Dilution Factor	: 1
Vial #	: 90
Injection Volume	: 1.00
Data File	: S:\Callum\DiKTa Project ESI\Proline addition purified.qgd
Org Data File	: S:\Callum\DiKTa Project ESI\Proline addition purified.qgd
Method File	: S:\standard method 1 for RTX-1 column 330 injection.qgm
Org Method File	: S:\standard method 1 for RTX-1 column 330 injection.qgm
Report File	:
Tuning File	: C:\GCMSsolution\System\Tune1\1 day after reinstall 09062021.qgt
Modified by	: Admin
Modified	: 22/05/2022 15:42:45

Chromatogram proline addition repurify S:\Callum\DiKTa Project ESI\Proline addition purified.qgd

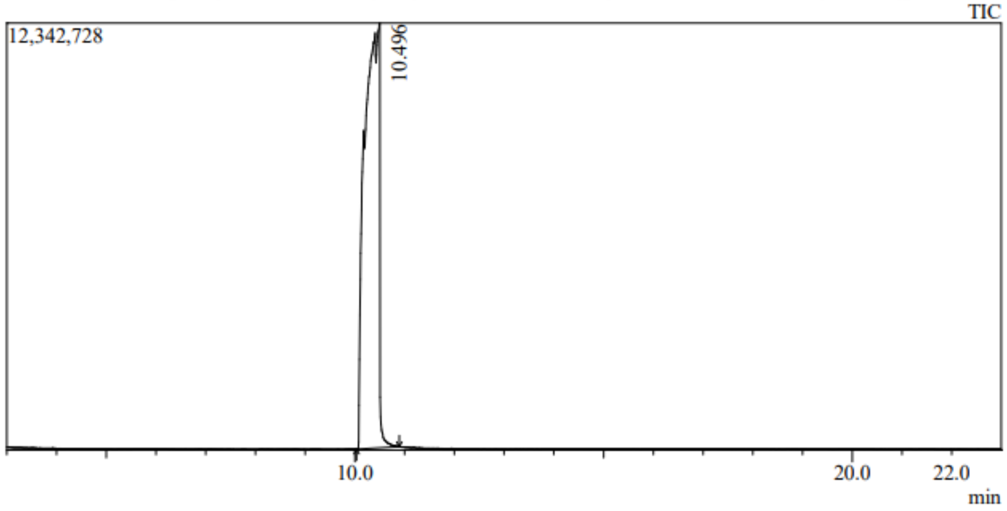

Peak Report TIC

Peak#	R.Time	Area	Area%	Height	A/H	Base m/z	Base Int.
1	10.496	254959655	100.00	12272994	20.77	91.10	4860908
		254959655	100.00	12272994			

Figure **S21**. GCMS trace of 2-(1-((benzyloxy)carbonyl)pyrrolidin-2- yl)succinate.

Sample Information

Analyzed by	: Admin
Analyzed	: 22/05/2022 14:45:58
Sample Type	: Unknown
Level #	: 1
Sample Name	: isobutyric acid addition
Sample ID	:
IS Amount	: [1]=1
Sample Amount	: 1
Dilution Factor	: 1
Vial #	: 89
Injection Volume	: 1.00
Data File	: S:\Callum\DiKTa Project ESI\isobutyric acid addition.qgd
Org Data File	: S:\Callum\DiKTa Project ESI\isobutyric acid addition.qgd
Method File	: S:\standard method 1 for RTX-1 column 330 injection.qgm
Org Method File	: S:\standard method 1 for RTX-1 column 330 injection.qgm
Report File	:
Tuning File	: C:\GCMSsolution\System\Tune1\1 day after reinstall 09062021.qgt
Modified by	: Admin
Modified	: 22/05/2022 15:08:59

Chromatogram isobutyric acid addition S:\Callum\DiKTa Project ESI\isobutyric acid addition.qgd

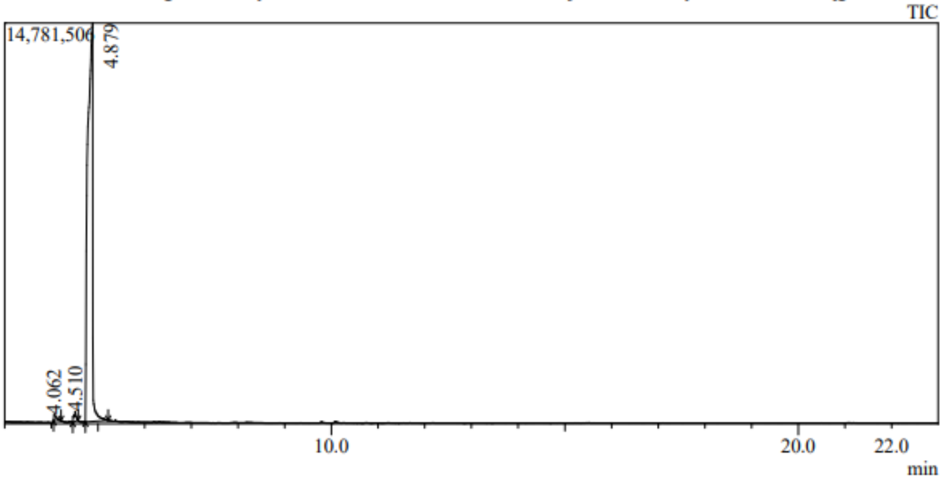

Peak Report TIC

Peak#	R.Time	Area	Area%	Height	A/H	Base m/z	Base Int.
1	4.062	819892	0.76	234569	3.50	127.10	45609
2	4.510	1166562	1.08	361327	3.23	157.15	29533
3	4.879	105839775	98.16	14693184	7.20	171.15	1173950
		107826229	100.00	15289080			

Figure **S22**. GCMS trace of diethyl 2-*iso*propylsuccinate.

Sample Information

Analyzed by	: Admin
Analyzed	: 22/05/2022 14:12:01
Sample Type	: Unknown
Level #	: 1
Sample Name	: propanoic acid addition
Sample ID	:
IS Amount	: [1]=1
Sample Amount	: 1
Dilution Factor	: 1
Vial #	: 88
Injection Volume	: 1.00
Data File	: S:\Callum\DiKTa Project ESI\propanoic acid addition mixture.qgd
Org Data File	: S:\Callum\DiKTa Project ESI\propanoic acid addition mixture.qgd
Method File	: S:\standard method 1 for RTX-1 column 330 injection.qgm
Org Method File	: S:\standard method 1 for RTX-1 column 330 injection.qgm
Report File	:
Tuning File	: C:\GCMSsolution\System\Tune1\1 day after reinstall 09062021.qgt
Modified by	: Admin
Modified	: 22/05/2022 14:35:02

Chromatogram propanoic acid addition S:\Callum\DiKTa Project ESI\propanoic acid addition mixture.qgd

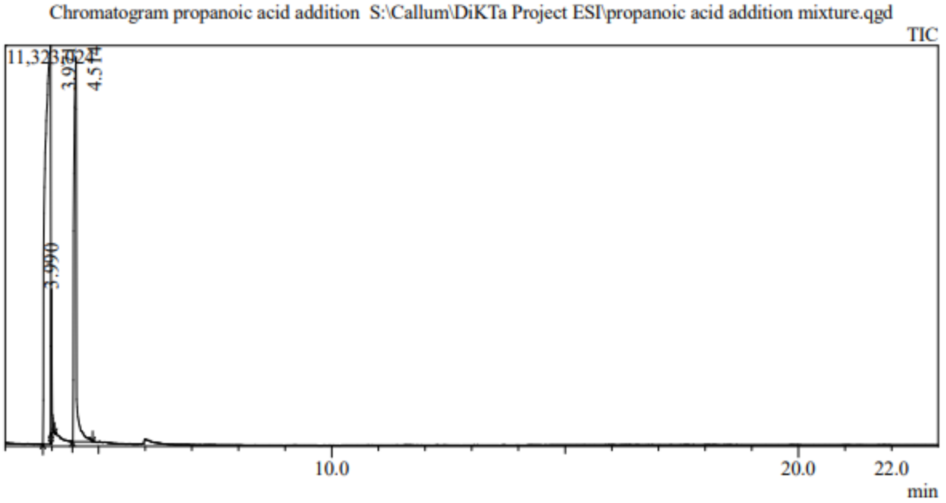

Peak Report TIC

Peak#	R.Time	Area	Area%	Height	A/H	Base m/z	Base Int.
1	3.971	90400922	66.04	11262446	8.03	99.05	2894135
2	3.990	3636559	2.66	4066018	0.89	101.05	243069
3	4.514	42844732	31.30	10876427	3.94	157.15	1274733
		136882213	100.00	26204891			

Figure **S23**. GCMS trace of mixture of diethyl 2-ethylsuccinate, diethyl succinate and diethyl maleate.

Sample Information

Analyzed by	: Admin
Analyzed	: 22/05/2022 16:27:42
Sample Type	: Unknown
Level #	: 1
Sample Name	: (Z)-stilbene
Sample ID	:
IS Amount	: [1]=1
Sample Amount	: 1
Dilution Factor	: 1
Vial #	: 92
Injection Volume	: 1.00
Data File	: S:\Callum\DiKTa Project ESI\cis stilbene repurified.qgd
Org Data File	: (Z)-stilbene
Method File	: S:\standard method 1 for RTX-1 column 330 injection.qgm
Org Method File	: S:\standard method 1 for RTX-1 column 330 injection.qgm
Report File	: (Z)-stilbene
Tuning File	: C:\GCMSsolution\System\Tune1\1 day after reinstall 09062021.qgt
Modified by	: Admin
Modified	: 22/05/2022 16:50:43

Chromatogram nickel coupling product repurified S:\Callum\DiKTa Project ESI\cis stilbene repurified.qgd

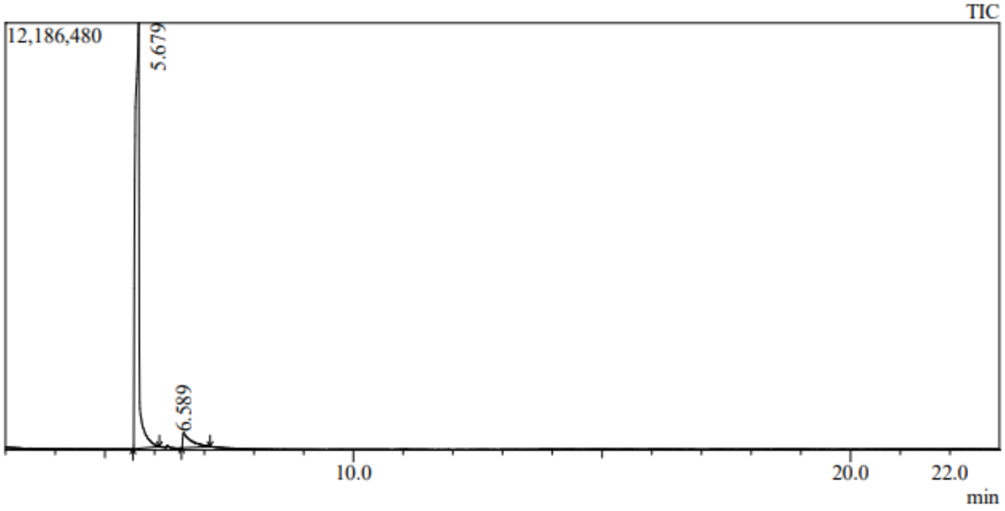

Peak Report TIC

Peak#	R.Time	Area	Area%	Height	A/H	Base m/z	Base Int.
1	5.679	68946238	93.23	12142660	5.68	180.15	1491045
2	6.589	5003915	6.77	433324	11.55	180.10	83791
		73950153	100.00	12575984			

Figure **S24**. GCMS trace of (*Z*)-Stilbene.

Sample Information

Analyzed by	: Admin
Analyzed	: 22/05/2022 13:38:08
Sample Type	: Unknown
Level #	: 1
Sample Name	: diisopropyl maleate
Sample ID	:
IS Amount	: [1]=1
Sample Amount	: 1
Dilution Factor	: 1
Vial #	: 87
Injection Volume	: 1.00
Data File	: S:\Callum\DiKTa Project ESI\diisopropyl maleate.qgd
Org Data File	: S:\Callum\DiKTa Project ESI\diisopropyl maleate.qgd
Method File	: S:\standard method 1 for RTX-1 column 330 injection.qgm
Org Method File	: S:\standard method 1 for RTX-1 column 330 injection.qgm
Report File	:
Tuning File	: C:\GCMSsolution\System\Tune1\1 day after reinstall 09062021.qgt
Modified by	: Admin
Modified	: 22/05/2022 14:01:09

Chromatogram diisopropyl maleate S:\Callum\DiKTa Project ESI\diisopropyl maleate.qgd

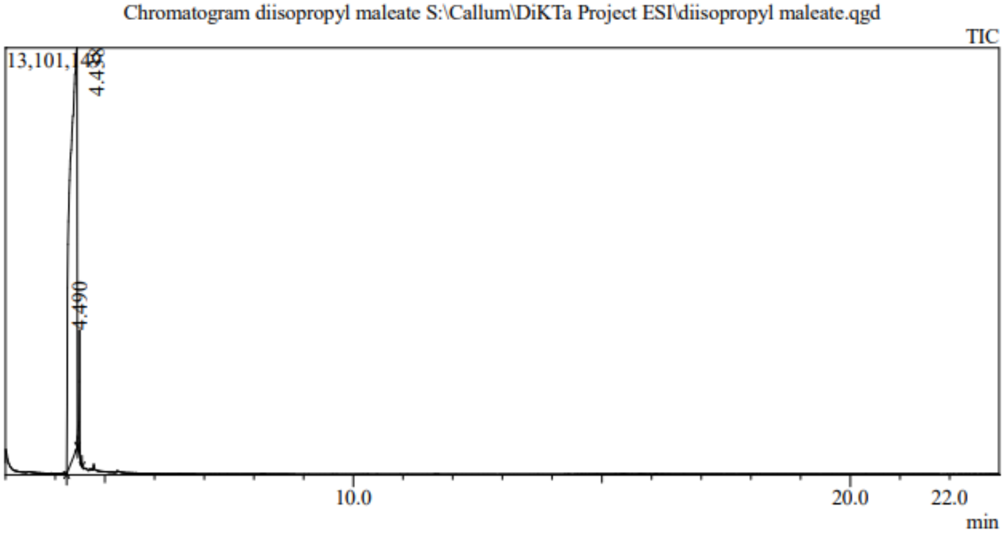

Peak Report TIC

Peak#	R.Time	Area	Area%	Height	A/H	Base m/z	Base Int.
1	4.438	120741195	96.87	12288862	9.83	99.10	4692516
2	4.490	3905847	3.13	3846654	1.02	141.15	466561
		124647042	100.00	16135516			

Figure **S25**. GCMS trace of di*iso*propyl maleate.

Sample Information

Analyzed by	: Admin
Analyzed	: 24/05/2022 15:27:28
Sample Type	: Unknown
Level #	: 1
Sample Name	: CP-III-nickel 2
Sample ID	:
IS Amount	: [1]=1
Sample Amount	: 1
Dilution Factor	: 1
Vial #	: 58
Injection Volume	: 1.00
Data File	: S:\Callum\DiKTa Project ESI\nickel 2.qgd
Org Data File	: S:\Callum\DiKTa Project ESI\nickel 2.qgd
Method File	: S:\standard method 1 for RTX-1 column 330 injection.qgm
Org Method File	: S:\standard method 1 for RTX-1 column 330 injection.qgm
Report File	:
Tuning File	: C:\GCMSsolution\System\Tune1\1 day after reinstall 09062021.qgt
Modified by	: Admin
Modified	: 24/05/2022 15:50:30

Chromatogram CP-III-nickel 2 S:\Callum\DiKTa Project ESI\nickel 2.qgd

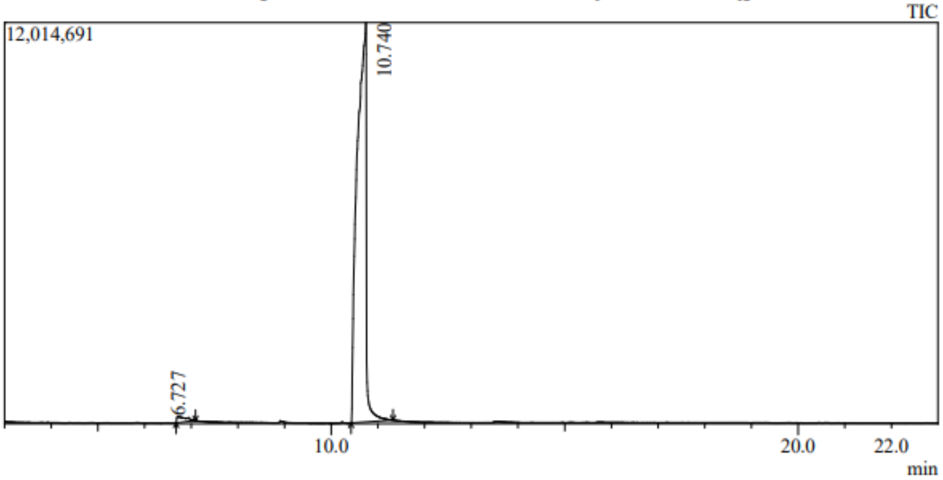

Peak Report TIC

Peak#	R.Time	Area	Area%	Height	A/H	Base m/z	Base Int.
1	6.727	2408240	1.45	177808	13.54	91.10	105338
2	10.740	164012230	98.55	11959016	13.71	91.15	4278136
		166420470	100.00	12136824			

Figure **S26**. GCMS trace of benzyl 2-(4-cyanophenyl)pyrrolidine-1-carboxylate.

Sample Information

Analyzed by	: Admin
Analyzed	: 22/05/2022 11:22:44
Sample Type	: Unknown
Level #	: 1
Sample Name	: N Boc Piperidine HAT
Sample ID	:
IS Amount	: [1]=1
Sample Amount	: 1
Dilution Factor	: 1
Vial #	: 83
Injection Volume	: 1.00
Data File	: S:\Callum\DiKTa Project ESI\HAT Product.qgd
Org Data File	: S:\Callum\DiKTa Project ESI\HAT Product.qgd
Method File	: S:\standard method 1 for RTX-1 column 330 injection.qgm
Org Method File	: S:\standard method 1 for RTX-1 column 330 injection.qgm
Report File	:
Tuning File	: C:\GCMSsolution\System\Tune1\1 day after reinstall 09062021.qgt
Modified by	: Admin
Modified	: 22/05/2022 11:45:46

Chromatogram N Boc Piperidine HAT S:\Callum\DiKTa Project ESI\HAT Product.qgd

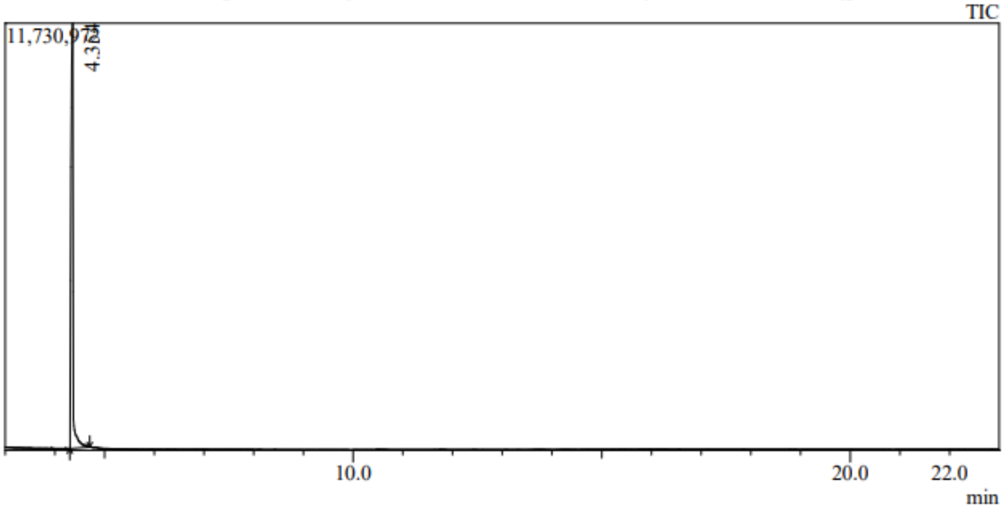

Peak Report TIC

Peak#	R.Time	Area	Area%	Height	A/H	Base m/z	Base Int.
1	4.354	33349253	100.00	11673253	2.86	57.10	2257545
		33349253	100.00	11673253			

Figure **S27**. GCMS trace of tert-butyl piperidine-1-carboxylate.

NMR Spectra

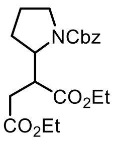

3a, ^1H, $CDCl_3$, 400 MHz

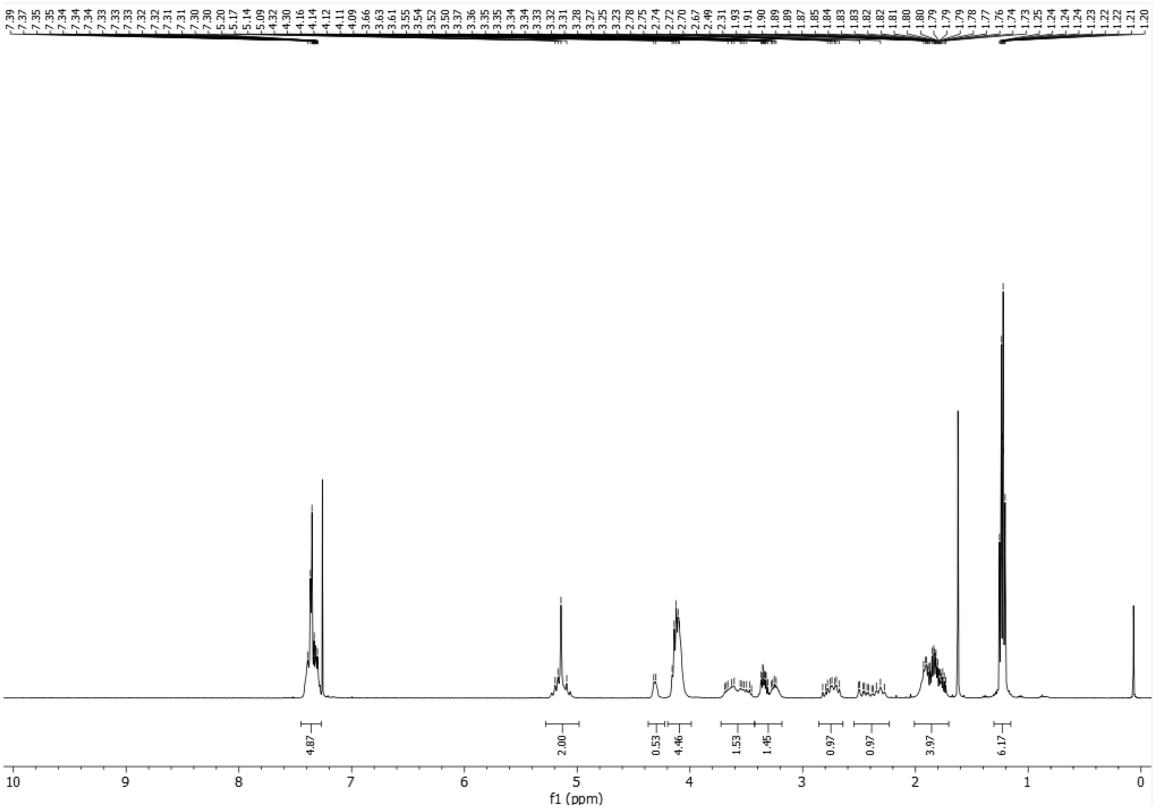

Figure **S28**. ^1H NMR spectrum of diethyl 2-(1-((benzyloxy)carbonyl)pyrrolidin-2-yl)succinate in $CDCl_3$.

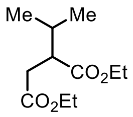

3b, ^{1}H, CDCl3, 500 MHz

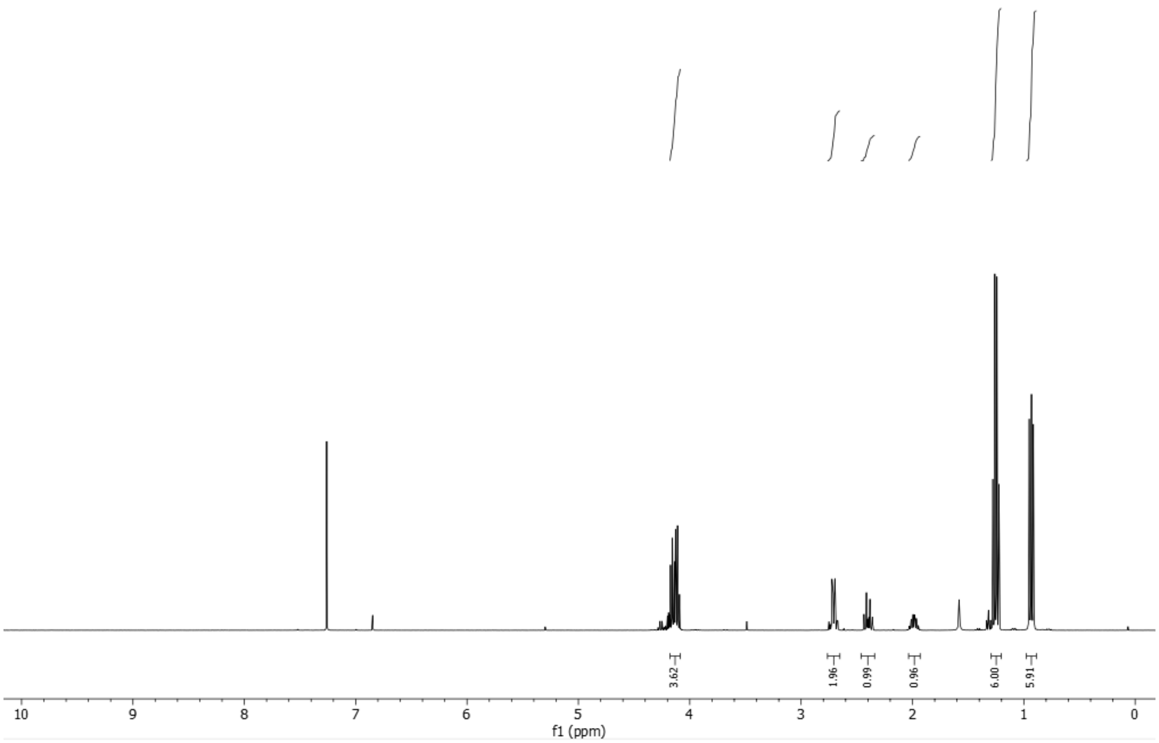

Figure **S29**. ^{1}H NMR spectrum of diethyl 2-*iso*propylsuccinate in $CDCl_3$.

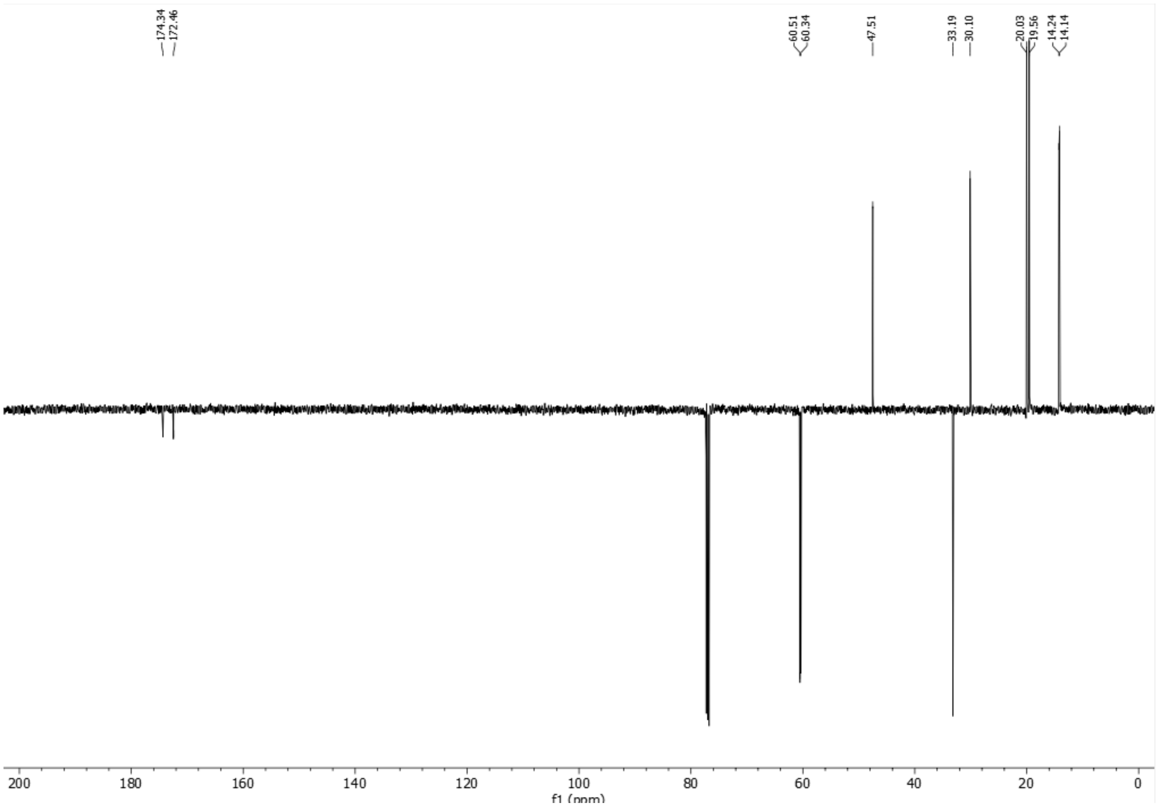

Figure **S30**. ^{13}C NMR spectrum of diethyl 2-*iso*propylsuccinate in $CDCl_3$.

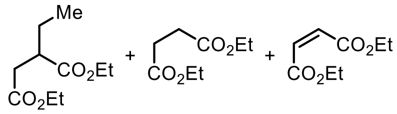

3c, ^{1}H, $CDCl_3$, 400 MHz

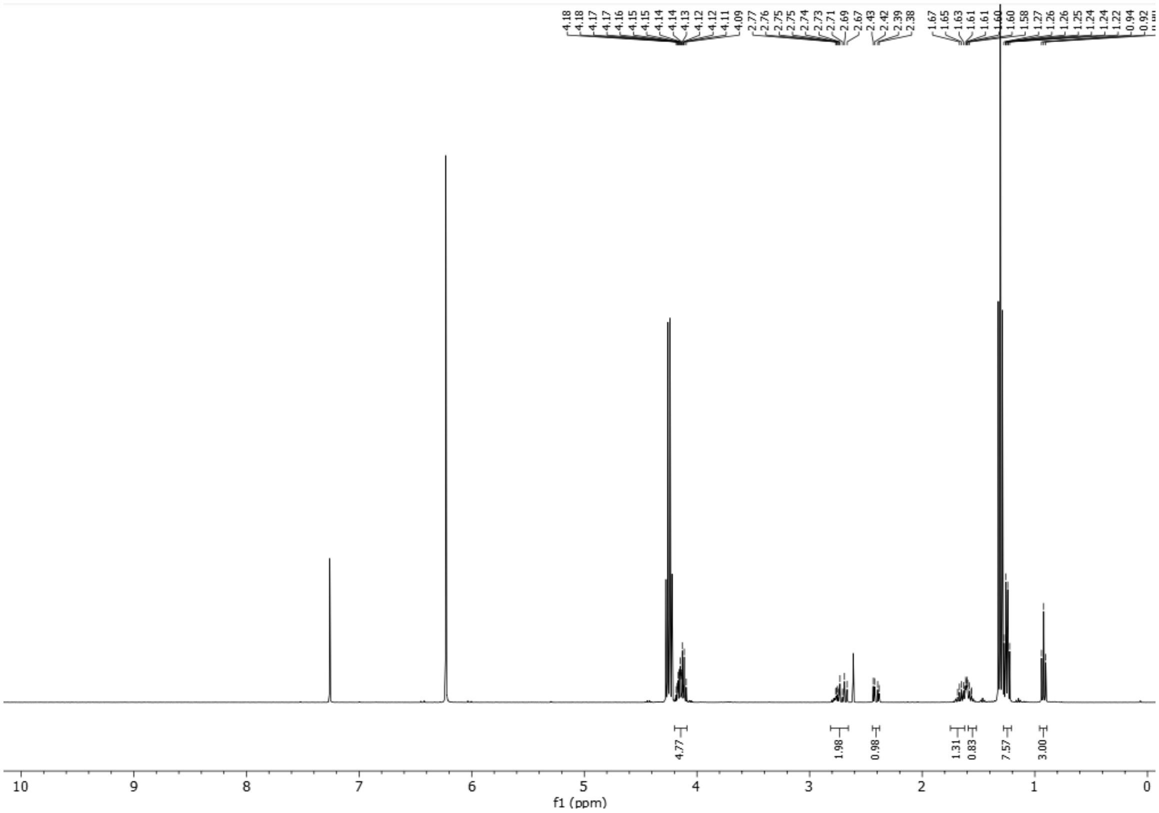

Figure **S31**. ^{1}H NMR spectrum of the mixture of diethyl 2-ethylsuccinate, diethyl succinate and diethyl maleate in $CDCl_3$.

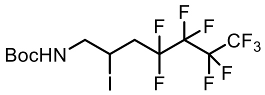

6a, ^{1}H, $CDCl_3$, 400 MHz

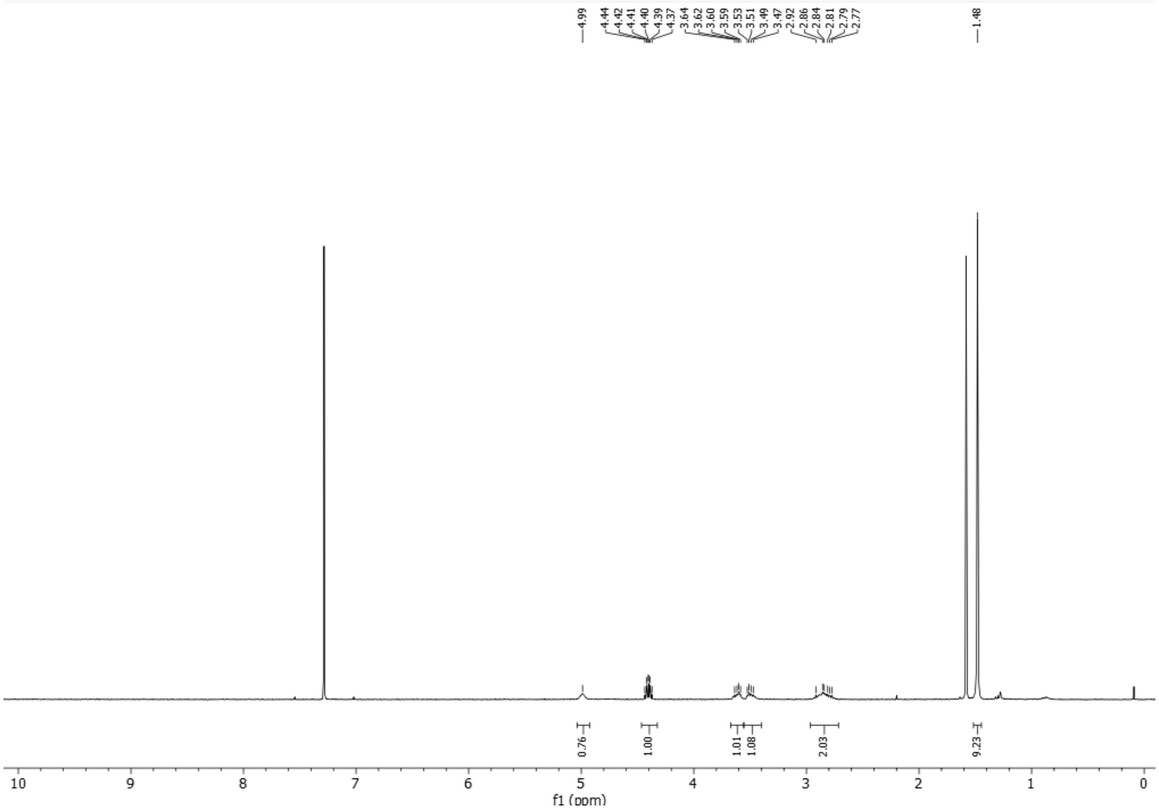

Figure **S32**. ^{1}H NMR spectrum of tert-butyl (4,4,5,5,6,6,7,7,7-nonafluoro-2-iodoheptyl)carbamate in $CDCl_3$.

6a, ^{19}F, $CDCl_3$, 376 MHz

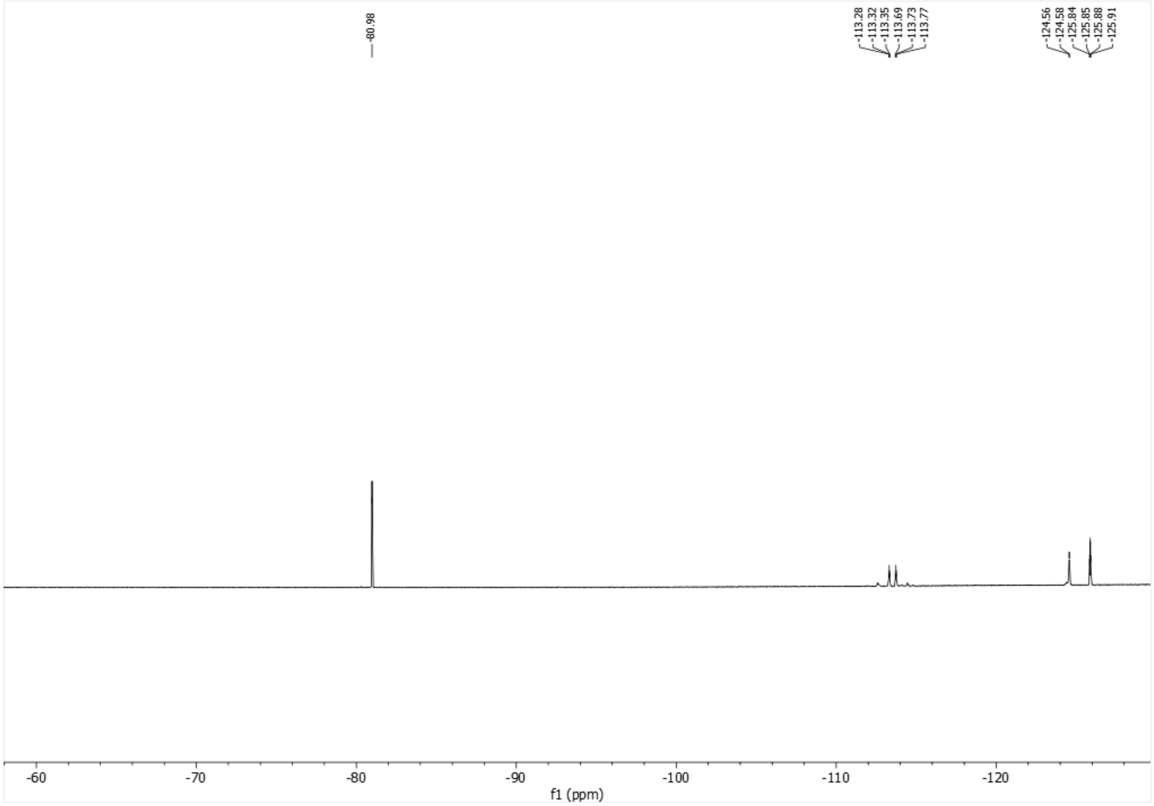

Figure **S33**. ^{19}F NMR spectrum of tert-butyl (4,4,5,5,6,6,7,7,7-nonafluoro-2-iodoheptyl)carbamate in $CDCl_3$.

6b, ^1H, $CDCl_3$, 400 MHz

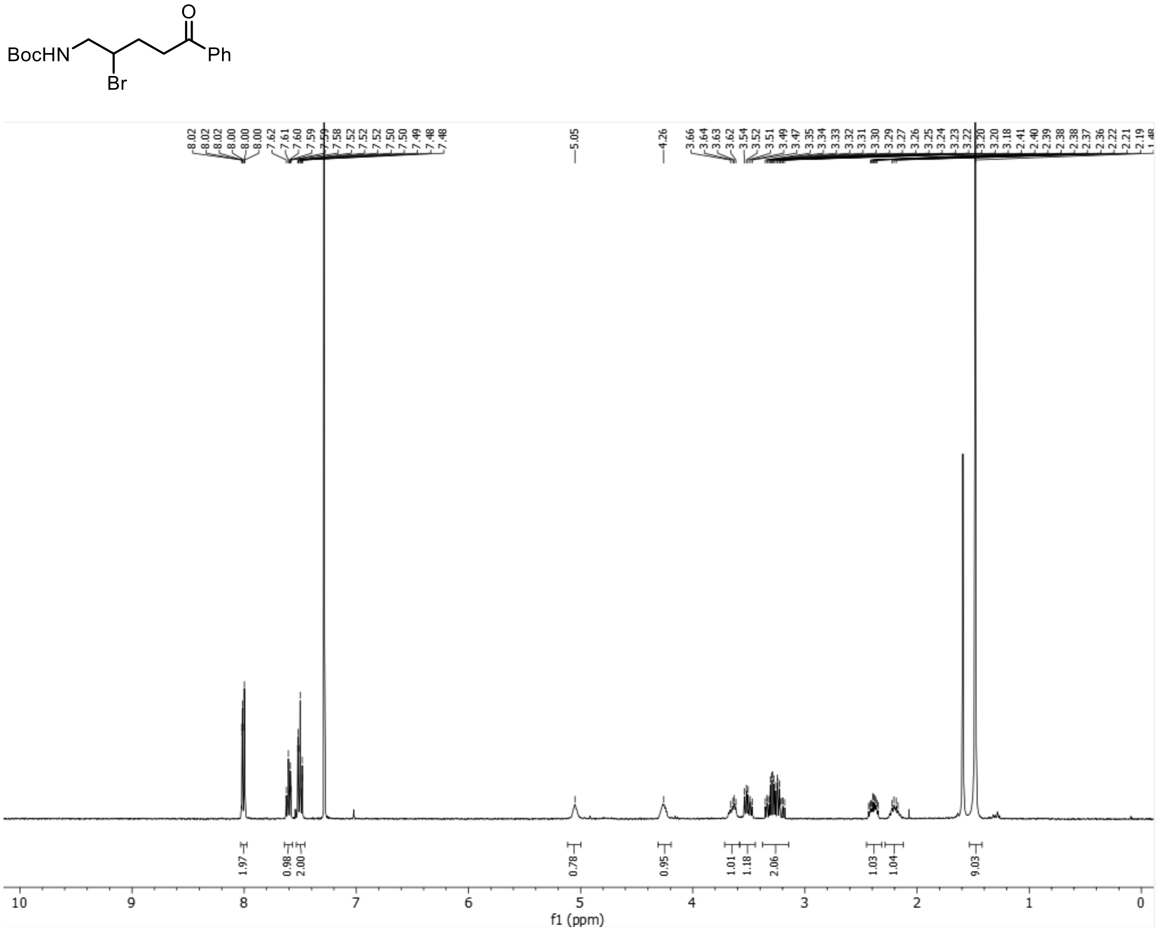

Figure **S34**. ^1H NMR spectrum of tert-butyl (2-bromo-5-oxo-5-phenylpentyl)carbamate in $CDCl_3$.

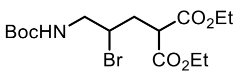

6c, ^{1}H, $CDCl_3$, 400 MHz

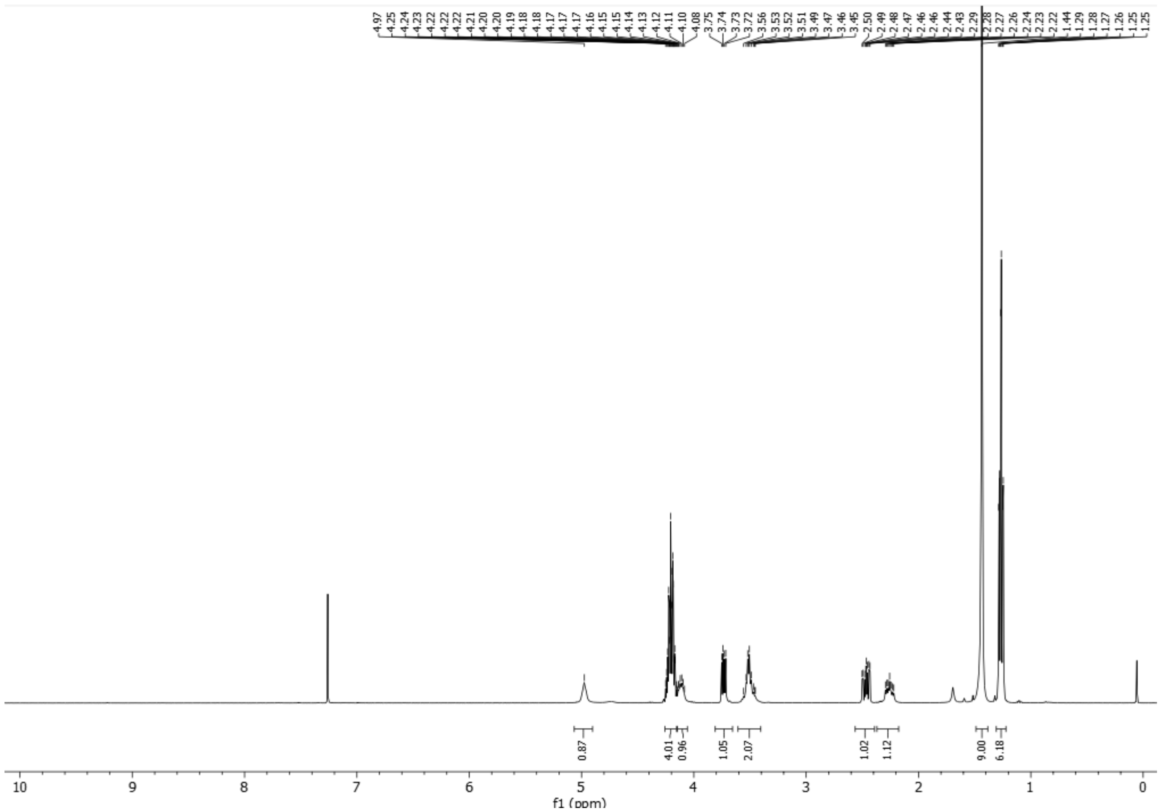

Figure **S35**. ^{1}H NMR spectrum of diethyl 2-(2-bromo-3-((tert-butoxycarbonyl)amino)propyl)malonate in $CDCl_3$.

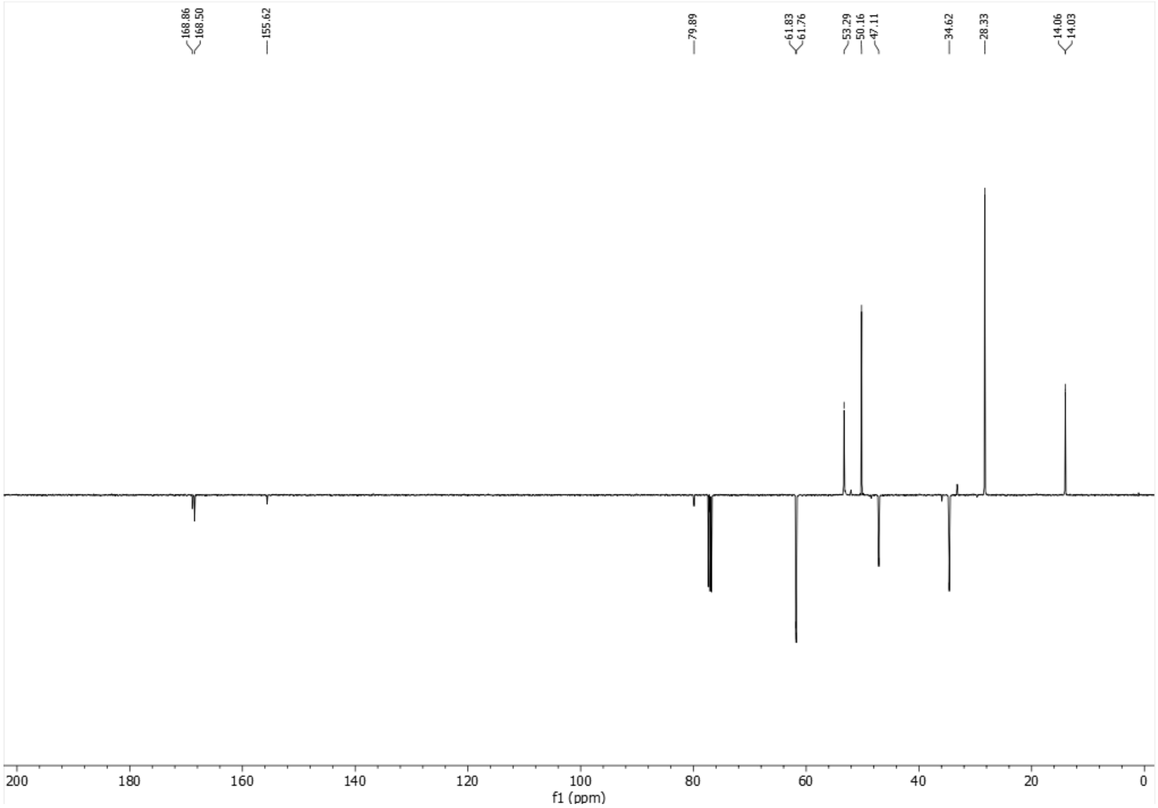

Figure **S36**. ^{13}C NMR spectrum of diethyl 2-(2-bromo-3-((tert-butoxycarbonyl)amino)propyl)malonate in $CDCl_3$.

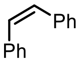

8a, ^{1}H, $CDCl_3$, 400 MHz

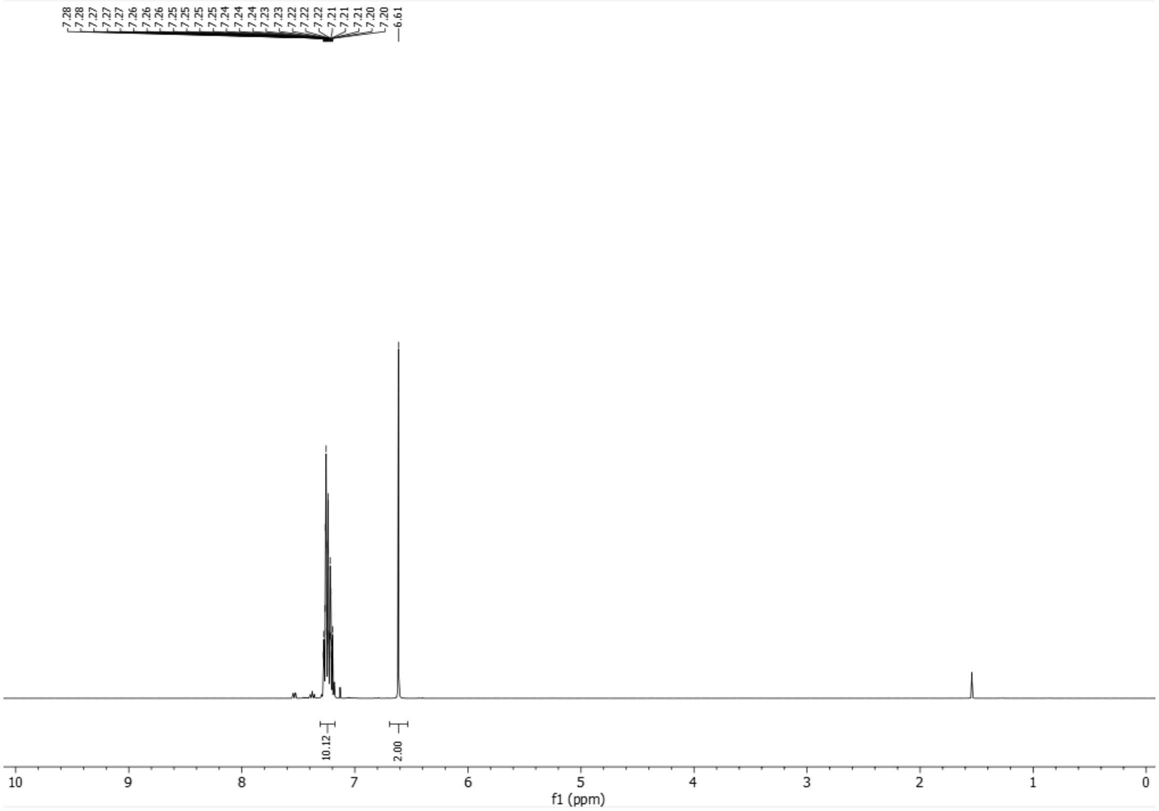

Figure **S37**. ^{1}H NMR spectrum of (*Z*)-Stilbene in $CDCl_3$.

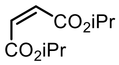

8b, ^{1}H, $CDCl_3$, 400 MHz

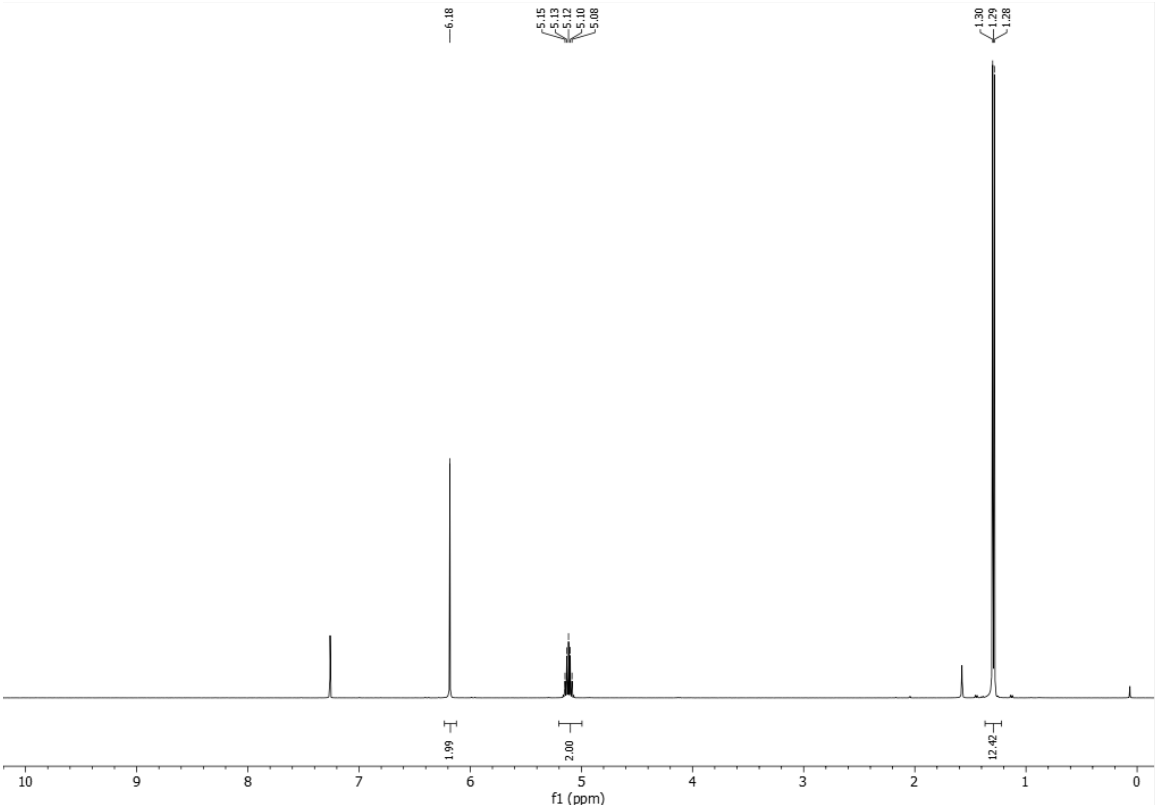

Figure **S38**. ^{1}H NMR spectrum of di*iso*propyl maleate in $CDCl_3$.

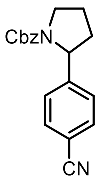

10, ^1H, $CDCl_3$, 400 MHz

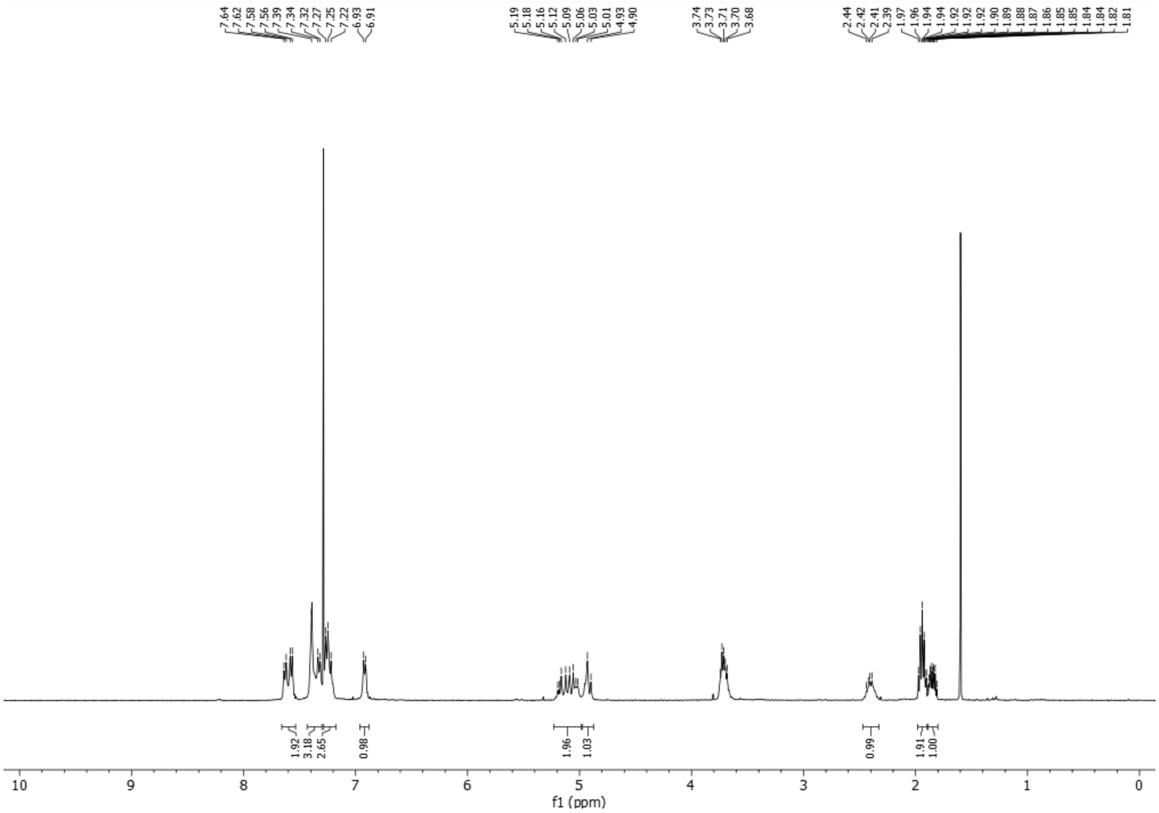

Figure **S39**. ^1H NMR spectrum of benzyl 2-(4-cyanophenyl)pyrrolidine-1-carboxylate in $CDCl_3$.

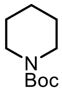

12, ^1H, $CDCl_3$, 400 MHz

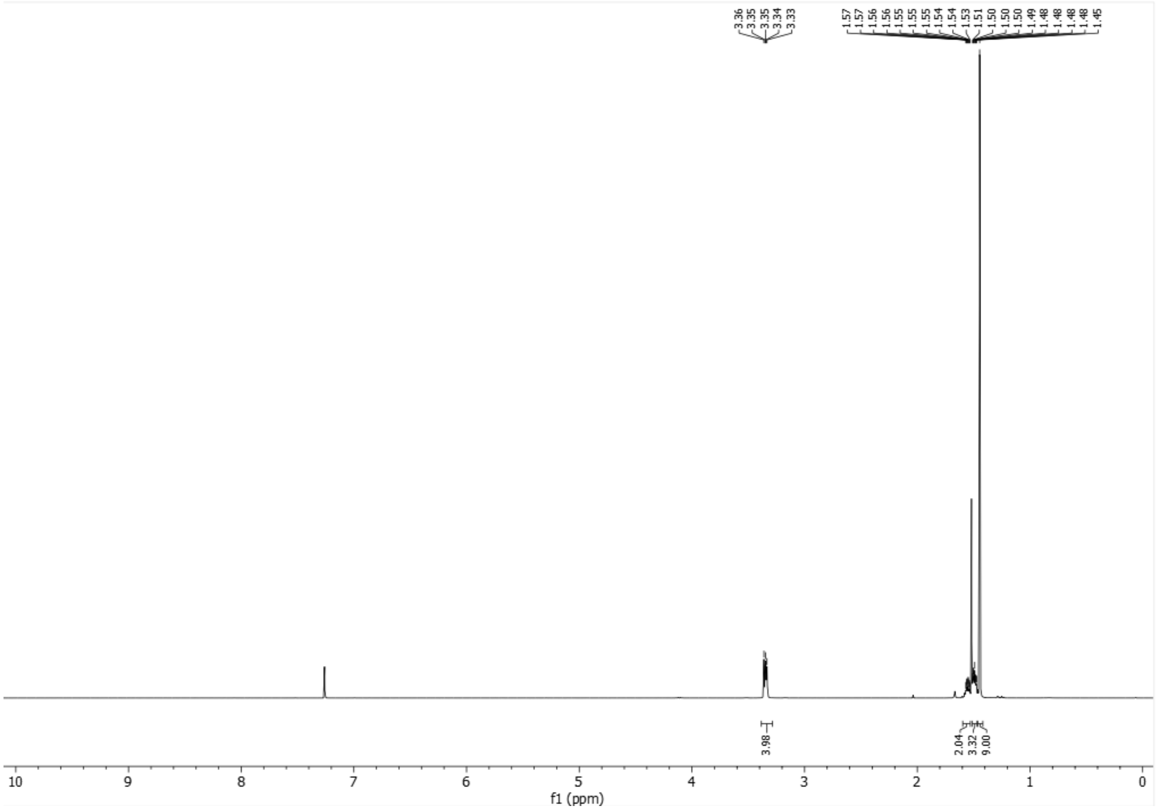

Figure **S40**. ^1H NMR spectrum of tert-butyl piperidine-1-carboxylate in $CDCl_3$.

References

[1] D. Subhas Bose, Mohd. Idrees, I. K. Todewale, N. M. Jakka, J. Venkateswara Rao, *Eur. J. Med. Chem.* **2012**, *50*, 27–38.

[2] B. Barré, L. Gonnard, R. Campagne, S. Reymond, J. Marin, P. Ciapetti, M. Brellier, A. Guérinot, J. Cossy, *Org. Lett.* **2014**, *16*, 6160–6163.

[3] Y. Tachibana, T. Masuda, M. Funabashi, M. Kunioka, *Biomacromolecules* **2010**, *11*, 2760–2765.

[4] J.-J. Dai, W.-M. Zhang, Y.-J. Shu, Y.-Y. Sun, J. Xu, Y.-S. Feng, H.-J. Xu, *Chem Commun* **2016**, *52*, 6793–6796.

[5] H. D. Pickford, J. Nugent, B. Owen, James. J. Mousseau, R. C. Smith, E. A. Anderson, *J. Am. Chem. Soc.* **2021**, *143*, 9729–9736.

[6] J. Luo, J. Zhang, *ACS Catal.* **2016**, *6*, 873–877.

[7] D. Hall, S. M. Suresh, P. L. dos Santos, E. Duda, S. Bagnich, A. Pershin, P. Rajamalli, D. B. Cordes, A. M. Z. Slawin, D. Beljonne, A. Köhler, I. D. W. Samuel, Y. Olivier, E. Zysman-Colman, *Adv. Opt. Mater.* **2020**, *8*, 1901627.

[8] N. G. Connelly, W. E. Geiger, *Chem. Rev.* **1996**, *96*, 877–910.

[9] B. Abadie, D. Jardel, G. Pozzi, P. Toullec, J.-M. Vincent, *Chem. – Eur. J.* **2019**, *25*, 16120–16127.

[10] Y. Ji, D. A. DiRocco, C. M. Hong, M. K. Wismer, M. Reibarkh, *Org. Lett.* **2018**, *20*, 2156–2159.

[11] S. Grotjahn, B. König, *Org. Lett.* **2021**, *23*, 3146–3150.

[12] K. Donabauer, M. Maity, A. L. Berger, G. S. Huff, S. Crespi, B. König, *Chem Sci* **2019**, *10*, 5162–5166.

[13] E. Speckmeier, T. G. Fischer, K. Zeitler, *J. Am. Chem. Soc.* **2018**, *140*, 15353–15365.

[14] F. Echalier, O. Constant, J. Bolte, *J. Org. Chem.* **1993**, *58*, 2747–2750.

[15] R. Beniazza, L. Remisse, D. Jardel, D. Lastécouères, J.-M. Vincent, *Chem Commun* **2018**, *54*, 7451–7454.

[16] M. Pirtsch, S. Paria, T. Matsuno, H. Isobe, O. Reiser, *Chem. – Eur. J.* **2012**, *18*, 7336–7340.

[17] J. Lu, B. Pattengale, Q. Liu, S. Yang, W. Shi, S. Li, J. Huang, J. Zhang, *J. Am. Chem. Soc.* **2018**, *140*, 13719–13725.

[18] N. Kaplaneris, A. Bisticha, G. N. Papadopoulos, D. Limnios, C. G. Kokotos, *Green Chem* **2017**, *19*, 4451–4456.

[19] Z. Zuo, D. W. C. MacMillan, *J. Am. Chem. Soc.* **2014**, *136*, 5257–5260.
